# Structural and mutational insights define ERMA as the ER Mg^2+^ ATPase and reservoir gatekeeper

**DOI:** 10.1126/sciadv.aef4971

**Published:** 2026-07-01

**Authors:** Manigandan Venkatesan, Michael L. Oldham, Ning Shi, Adhishree Chidambaram, Neelanjan Vishnu, Abitha K. Madesh, Kristen Bentz, Peter B. Stathopulos, Ravi C. Kalathur, Youxing Jiang, Muniswamy Madesh

**Affiliations:** ^1^ Department of Medicine, University of Texas Health San Antonio, San Antonio, TX, USA.; ^2^ Center for Mitochondrial Medicine, University of Texas Health San Antonio, San Antonio, TX, USA.; ^3^ Department of Structural Biology, St Jude Children’s Research Hospital, Memphis, TN, USA.; ^4^ Department of Physiology, The University of Texas Southwestern Medical Center, Dallas, TX, USA.; ^5^ Department of Biophysics, The University of Texas Southwestern Medical Center, Dallas,TX, USA.; ^6^ Howard Hughes Medical Institute, Chevy Chase, MD, USA.; ^7^ Department of Physiology and Pharmacology, Western University, London, ON N6A 5C1, Canada.

## Abstract

Magnesium (Mg^2+^) is the most abundant divalent cation in cells, yet the mechanisms mediating its organellar transport remain poorly defined. We identify endoplasmic reticulum (ER) Mg^2+^ adenosine triphosphatase (ATPase) (ERMA) as the transporter that drives Mg^2+^ uptake into the ER lumen, establishing the ER as a bi-ionic intracellular reservoir. MagFRET biosensors targeted to the ER demonstrate that ERMA mediates dynamic ER Mg^2+^ storage and robust adenosine 5′-triphosphate–dependent Mg^2+^ uptake reaching 15 to 30 millimolar. Cryo–electron microscopy structures of human and mouse ERMA reveal a P-type ATPase fold with an unwound transmembrane 4 (TM4) that coordinates Mg^2+^ via the unique PILP backbone and the TM5 residue Q1110, whose mutation markedly impairs ERMA-mediated Mg^2+^ uptake. Functional reconstitution of domain mutants, ERMA-SERCA chimeras, and pathogenic variants confirm ERMA as an ER-resident Mg^2+^ pump and gatekeeper of ER Mg^2+^ ionic equilibrium.

## Teaser

An ATP-powered ER Mg^2+^ pump, ERMA, establishes the ER/SR as a bi-ionic reservoir shaping cellular ion balance.

## INTRODUCTION

P-type adenosine triphosphatases (ATPases) constitute a large and evolutionarily conserved superfamily of primary active transporters that couple adenosine 5′-triphosphate (ATP) hydrolysis to the vectorial translocation of diverse substrates across biological membranes (*1*). Subfamilies within this group have evolved to selectively transport Na^+^/K^+^, H^+^, Ca^2+^, Cu^2+^, Zn^2+^, and metabolites, thereby establishing an electrochemical gradient, preserving intracellular ion compartmentalization, and maintaining membrane integrity (*1*–*10*). Structural studies of these pumps have revealed conserved mechanistic principles, in which cytosolic actuator (A), phosphorylation (P), and nucleotide-binding (N) domains couple ATP hydrolysis to substrate translocation through selective transmembrane (TM)–binding sites. In addition, each subgroup has evolved unique adaptations to achieve selectivity for distinct substrates. Despite extensive structural and mechanistic studies on sodium/potassium-transporting ATPase (Na^+^/K^+^), Ca^2+^, and H^+^ ATPases, comparatively little is known about how P-type ATPases achieve selectivity and catalysis for magnesium (Mg^2+^), an ion with distinct hydration and coordination chemistry compared to other biologically relevant cations (*11*–*25*). Among biologically essential cations, Mg^2+^ is distinguished by its abundance and significance to cellular physiology (*25*–*27*). Within cells, Mg^2+^ is crucial for stabilizing ribosomes, supporting cotranslational protein translocation, and enabling ATP-dependent enzymatic reactions, where it acts as an obligate cofactor for nucleotide binding and hydrolysis. Perturbations in intracellular Mg^2+^ (_i_Mg^2+^) dynamics have been linked to loss of translational fidelity, triggering of unfolded protein response, and several human pathologies, underscoring the importance of tight compartmental regulation (*25*, *27*–*31*). Yet, compared to other ions, the structural and mechanistic basis of Mg^2+^ transport remains poorly understood. In bacteria, P-type ATPases MgtA and MgtB mediate Mg^2+^ uptake under limiting conditions, and structural work on MgtA has revealed the canonical P-type fold and Mg^2+^ selective features (*32*–*35*). However, these bacterial pumps function exclusively at the cytoplasmic membrane and are specialized for adaptation to nutrient limitation, whereas eukaryotic cells must additionally regulate Mg^2+^ within intracellular organelles to sustain translation, ATP-dependent folding, and signaling processes. Thus, although bacterial Mg^2+^ pumps have provided critical insights into ion selectivity, they do not recapitulate the specialized requirements of organellar compartmentalization, leaving unresolved eukaryotic Mg^2+^ handling, particularly in the endoplasmic reticulum (ER) (*36*, *37*).

The ER compartment not only serves as the principal Ca^2+^ reservoir but also requires tightly regulated Mg^2+^ homeostasis to support ribosome integrity, protein translation, ATP utilization, and the folding of secretory proteins (*38*–*40*). Although Ca^2+^ has been established as the dominant signaling ion of the ER, Mg^2+^ dynamically modulates Ca^2+^ channel function and buffering capacity, positioning it as an essential regulator of ER function. We recently revealed that l-lactate triggers Mg^2+^ release from the ER followed by Mg^2+^ uptake by the mitochondria. During this process, lactate-induced rapid _ER_Mg^2+^ depletion was followed by a robust mitochondrial Mg^2+^ (_m_Mg^2+^) uptake (*25*). Our subsequent bioinformatics, targeted small interfering RNA screening, and AlphaFold2 structure prediction revealed that an uncharacterized TMEM94 [ER Mg^2+^ ATPase (ERMA)] protein resides in the ER permeates Mg^2+^ and refills the luminal compartment (*24*). Just as sarco/ER Ca^2+^-ATPase (SERCA) resolved the enigma of ER Ca^2+^ sequestration, the identification of an ER-localized Mg^2+^ pump addresses a longstanding gap in our understanding of compartmentalized Mg^2+^ regulation. While the molecular machinery underlying ER Ca^2+^ flux has been elucidated through the study of SERCAs, inositol 1,4,5-trisphosphate (IP_3_) receptors, and ryanodine receptors, the mechanism of an ER-resident Mg^2+^ transporter has remained elusive. To date, no high-resolution structure of a eukaryotic Mg^2+^ P-type ATPase has been available, leaving fundamental questions unanswered regarding how this essential class of transporters achieves Mg^2+^ specificity.

AlphaFold2 modeling suggested that ERMA adopts the overall architecture of P-type ATPases but incorporates distinctive motifs, including a noncanonical phosphorylation loop (DKQGIL) and a conserved GMN motif (*24*). Nonetheless, the structural basis by which ERMA catalyzes Mg^2+^ transport, and how its features confer substrate specificity, has remained elusive. This knowledge gap is particularly consequential as emerging evidence links _ER_Mg^2+^ dynamics to translational fidelity, proteostasis, and stress adaptation, and mutations in ERMA have been associated with human pathologies (*41*). Here, we combine cryo–electron microscopy (cryo-EM) and biochemical reconstitution to establish ERMA as a structurally resolved ER-resident Mg^2+^ ATPase. We show that ERMA retains the catalytic machinery of the superfamily while exhibiting conserved 10-TM adaptations required for Mg^2+^ recognition and transport. Functional assays in proteoliposomes and permeabilized cells confirm that ERMA directly mediates ATP-dependent Mg^2+^ uptake into the ER lumen. Furthermore, pathogenic and site-specific mutations reveal structural elements essential for catalysis and ion coordination.

## RESULTS

### MagFRET sensor calibration reveals the ER as a major _i_Mg^2+^ reservoir

**Fig. 1.**
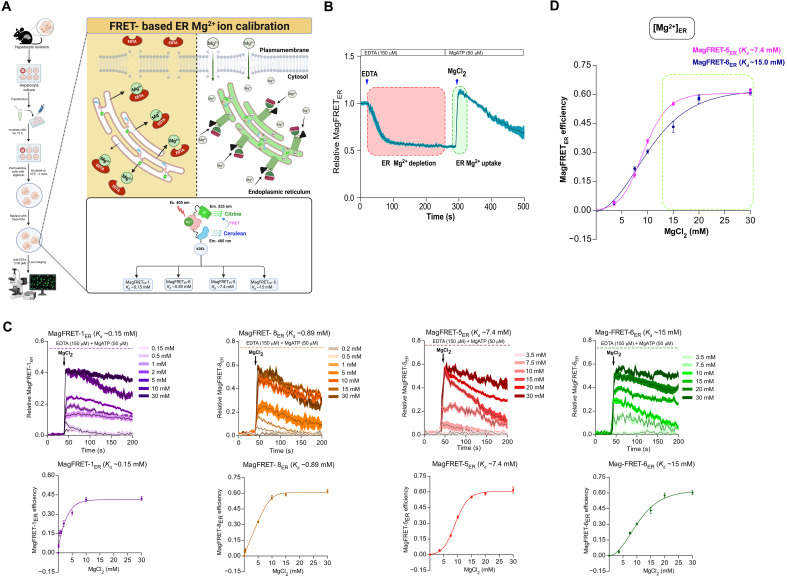
MagFRET-based calibration reveals high luminal ER Mg^2+^ concentration and active ER Mg^2+^ uptake. (**A**) Schematic illustrates the experimental workflow and principle of the _ER_Mg^2+^ calibration assay. Primary murine hepatocytes were isolated and transfected with MagFRET_ER_ sensors variants with distinct dissociation constants (*K*_d_) enabling detection across a broad dynamic range of free ER luminal Mg^2+^. This calibration framework was used throughout the study to quantify _ER_Mg^2+^ uptake in permeabilized hepatocytes and MEFs. Created in BioRender. Venkatesan, M. (2026) https://BioRender.com/8723oiv. h, hours. (**B**) Representative mean trace of MagFRET_ER_ response in murine hepatocytes showing dynamic changes in ER luminal Mg^2+^ levels. Upon addition of EDTA (blue arrow), a rapid loss of MagFRET signals reflects _ER_Mg^2+^ depletion (red box). After reaching a baseline signals, subsequent addition of 50 μM Mg-ATP and a bolus of 5 mM MgCl_2_ elicited a robust increase in the MagFRET_ER_ signal, corresponding to _ER_Mg^2+^ uptake (green box). Mean ± SEM. (*n* = 4 independent experiments). (**C**) Primary murine hepatocytes were transfected with MagFRET_ER_ sensors of varying *K*_d_s. Normalized mean traces (top row) and corresponding calibration curves (bottom row) of four MagFRET_ER_ variants. Following EDTA-mediated _ER_Mg^2+^ depletion, the cells were exposed to dose-dependent MgCl_2_ concentrations (0.5 to 30 mM). MagFRET-1_ER_ (*K*_d_ ~0.15 mM, purple), MagFRET-8_ER_ (*K*_d_ ~0.89 mM, orange), MagFRET-5_ER_ (*K*_d_ ~7.4 mM, red), and MagFRET-6_ER_ (*K*_d_ ~15 mM, green) exhibited distinct sensitivities in the presence of 50 μM Mg-ATP. Dose-response relationships were fit using nonlinear regression to define sensor performance. Data are presented as means ± SEM from *n* = 3 independent experiments. (**D**) Overlay of MagFRET-5_ER_ and MagFRET-6_ER_ calibration curves. Both sensors reach saturation at elevated MgCl_2_, with half-maximal responses near their respective *K*_d_ values. The plateau at 15–30 mM MgCl_2_ indicates free luminal [Mg^2+^]_ER_ resides within this concentration window.

Although studies have estimated the concentration of intracellular bound Mg^2+^ to be 15 to 30 mM, the spatial distribution, compartmentalization, and dynamic regulation of the free ionized Mg^2+^ pool within distinct organelles remain poorly characterized (*26*, *39*, *42*). This knowledge gap is particularly pronounced in the ER, an organelle where ion composition critically influences protein folding, lipid synthesis, Ca^2+^ signaling, and interorganellar communication. To address this, we developed a Magnesium Forster resonance energy transfer (MagFRET)-based assay to monitor _ER_Mg^2+^ dynamics. Primary murine hepatocytes were transfected with the ER-targeted MagFRET (MagFRET_ER_) sensor, enabling sensitive detection of luminal [Mg^2+^], providing a direct readout of _ER_Mg^2+^ (*43*). To measure _ER_Mg^2+^ uptake, hepatocytes were permeabilized with digitonin (40 μg/ml) containing intracellular medium (ICM) briefly (*24*). Permeabilized cells bathed in ICM containing EDTA (150 μM) triggered a rapid reduction of the MagFRET_ER_ signal, reaching a stable baseline at ~200 s. Subsequent suspension with 50 μM magnesium-ATP (Mg-ATP) followed by the addition of extracellular MgCl_2_ (10 mM) increased the MagFRET signal, demonstrating that the ER membrane actively refills Mg^2+^ and suggesting the ER as a dynamic reservoir (Fig. 1, A and B). To estimate the MagFRET-based measurements of ER luminal [Mg^2+^] in the permeabilized cell system, we assessed retention of the MagFRET-1_ER_ sensor in intact, permeabilized, and EDTA-chelated permeabilized cells. Confocal imaging revealed that MagFRET-1_ER_ signal was largely retained following permeabilization (300 s) (fig. S1, A to C). In contrast, subsequent treatment with EDTA following permeabilization resulted in a marked reduction in MagFRET signal without evidence of spatial dispersion of cerulean (cyan fluorescent protein) and citrine (yellow fluorescent protein) signals (fig. S1C). In intact cells, MagFRET-1_ER_ signals remained stable over the recording period, reflecting steady-state luminal Mg^2+^ levels (fig. S1, D and E). Upon digitonin permeabilization, only a modest change in baseline FRET was observed, indicating that membrane permeabilization alone does not induce sensor loss or ER rupture. However, addition of EDTA to permeabilized cells triggered a rapid and sustained decrease in MagFRET-1 signal, reaching a new baseline within ~100 to 150 s (fig. S1, D and E). Quantitative analysis of ΔMagFRET-1 signals demonstrated no substantial difference between intact and permeabilized conditions (fig. S1F).

To further validate the ERMA-mediated Mg^2+^ uptake in the permeabilized system, we next assessed Mg^2+^ uptake under defined MgCl_2_ and ATP conditions. In cells presupplemented with Mg-ATP, the subsequent addition of MgCl_2_ elicited a rapid increase in MagFRET-1_ER_ signal (fig. S1G, green trace). In contrast, in the absence of prior Mg-ATP, addition of MgCl_2_ alone failed to induce any detectable FRET response in _ER_Mg^2+^, demonstrating that ERMA-mediated Mg^2+^ uptake requires Mg-ATP (fig. S1G). Under this condition, later addition of Mg-ATP triggered a gradual and steady increase in MagFRET-1_ER_ signal, indicating that Mg-ATP is required to initiate _ER_Mg^2+^ transport (fig. S1G). Quantification of ΔMagFRET-1_ER_ signals confirmed that robust _ER_Mg^2+^ accumulation occurs only in the presence of both Mg^2+^ and Mg-ATP, whereas MgCl_2_ alone does not produce a significant response (fig. S1, H to I). These results demonstrate that _ER_Mg^2+^ uptake is ATP dependent and that the temporal availability of Mg-ATP critically influences transport efficiency and kinetics. Collectively, these data demonstrate that digitonin permeabilization preserves ER membrane integrity and retains MagFRET sensors within the ER lumen, while also establishing that the measured signals reflect an ATP-dependent _ER_Mg^2+^ transport mechanism rather than passive ion flux or probe redistribution. Together, the permeabilized MagFRET_ER_ assay is suitable for _ER_Mg^2+^ uptake and demonstrates that the ER reservoir is readily exchangeable in response to changes in cytosolic [Mg^2+^].

To determine the concentration of free magnesium ions [Mg^2+^]_ER_ within the ER, we performed a calibration using a panel of MagFRET_ER_ sensors with distinct dissociation constants (*K*_d_). These sensors, MagFRET-1_ER_ (*K*_d_ ~0.15 mM), MagFRET-8_ER_ (*K*_d_ ~0.89 mM), MagFRET-5_ER_ (*K*_d_ ~7.4 mM), and MagFRET-6_ER_ (*K*_d_ ~15 mM), span a broad dynamic range of affinities, enabling sensitive detection of Mg^2+^ across physiological concentrations (*43*). Upon stepwise addition of MgCl_2_ at various concentrations, MagFRET sensors exhibited robust and concentration-dependent increases in FRET ratio, followed by sustained signals throughout the recording period (Fig. 1C, top). Low-affinity sensors such as MagFRET-5_ER_ and MagFRET-6_ER_ required higher MgCl_2_ concentrations (≥7.5 mM) to elicit detectable responses, whereas the high-affinity MagFRET-1_ER_ responded sharply even at submillimolar concentrations (0.15 to 0.5 mM). MagFRET-8_ER_, with intermediate affinity (*K*_d_ ~0.89 mM), saturated at midmillimolar MgCl_2_, bridging the range between MagFRET-1_ER_ and the lower-affinity constructs. Dose-response analysis revealed distinct binding curves consistent with the sensors’ calibrated *K*_d_ values (*43*). MagFRET-1_ER_ achieved half-maximal response at ~0.15 mM MgCl_2_ and reached saturation near 10 mM, confirming its utility in detecting low-end [Mg^2+^]_ER_. MagFRET-8_ER_ saturated above ~5 mM MgCl_2_, while MagFRET-5_ER_ and MagFRET-6_ER_ required progressively higher concentrations, saturating at approximately 15 and 20 mM, respectively (Fig. 1C, bottom). Our comprehensive analysis using the lower-affinity MagFRET-5_ER_ (*K*_d_ ~7.4 mM) and MagFRET-6_ER_ (*K*_d_ ~15 mM) revealed that the [Mg^2+^] within the ER lies in the higher millimolar range. Both sensors exhibited characteristic sigmoidal binding curves upon titration with MgCl_2_, with half-maximal responses aligning with their respective *K*_d_ values (Fig. 1D). The overlapping dynamic range of these two sensors provided a critical window for estimating [Mg^2+^]_ER_. Notably, the overlapping dynamic ranges of MagFRET-5_ER_ and MagFRET-6_ER_ provided an interpretable window, allowing us to estimate that free [Mg^2+^]_ER_ is between 15 and 30 mM under a permeabilized condition that is closely linked to the cellular bound [Mg^2+^] (Fig. 1D). These data indicate that the ER functions as a reservoir for Mg^2+^, with concentrations substantially higher than those typically found in the cytosol (~0.5 mM) (*26*, *29*). Having performed _ER_Mg^2+^ calibration, we next applied this framework to directly test whether ERMA constitutes the functional route for cytosol-to-ER Mg^2+^ transport.

To investigate the functional requirement of ERMA in _ER_Mg^2+^ uptake, we generated a conditional *Erma^fl/fl^* mouse allele by introducing *loxP* sites flanking critical exons following FLP Recognition Target (*FRT)*-mediated removal of the neomycin cassette, enabling hepatocyte-specific gene deletion upon Adenovirus expressing improved Cre recombinase (Ad-iCre) recombination (fig. S2A). Polymerase chain reaction (PCR) genotyping confirmed precise allele configuration and the successful derivation of *Erma^+/+^, Erma^+/fl^,* and *Erma^fl/fl^* mice (fig. S2B). Primary hepatocytes isolated from *Erma^fl/fl^* animals expressed robust levels of ERMA protein; however, following 72 hours of Ad-iCre treatment, ERMA protein was depleted while Serca2A and β actin levels remained unchanged (fig. S2C). To confirm the ERMA requirement for _ER_Mg^2+^ uptake, primary hepatocytes isolated from *Erma^fl/fl^* mice were transduced with Ad-iCre to induce ERMA deletion, followed by transfection with MagFRET-6_ER_ to monitor ER luminal Mg^2+^ uptake. Permeabilized hepatocytes were exposed to various concentrations of MgCl_2_ (7.5, 15, or 30 mM). In *Erma^fl/fl^* hepatocytes, the MagFRET-6_ER_ signal was robust and concentration-dependent _ER_Mg^2+^ uptake (fig. S2, D and E). The MagFRET signal in *Erma^fl/fl^* hepatocytes is comparable to that of wild-type (WT) hepatocytes (fig. S2E and Fig. 1C). In contrast, *Erma^fl/fl^* + Ad-iCre–transduced hepatocytes did not show any detectable change in luminal Mg^2+^ FRET signal (fig. S2, D and E). Together, these data demonstrate that ERMA is a major route for _ER_Mg^2+^ uptake and stores high concentrations of free Mg^2+^ that are readily exchangeable during ER depletion or uptake mechanisms.

### Reconstituted human ERMA drives ATP-dependent Mg^2+^ uptake, while the loss-of-function mutant fails to transport Mg^2+^

**Fig. 2.**
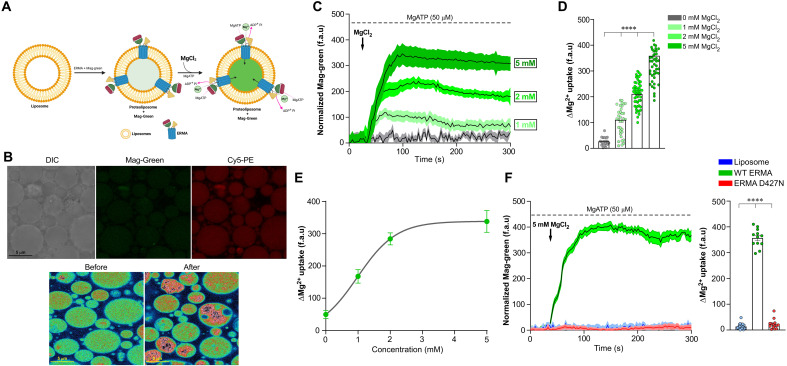
Reconstitution of ERMA-mediated Mg^2+^ transport in proteoliposomes. (**A**) Schematic representation of the liposome-based Mg^2+^ flux assay to assess ERMA Mg^2+^ transport activity. Created in BioRender. Venkatesan, M. (2026) https://BioRender.com/13orfli. (**B**) Representative confocal microscopy images of proteoliposomes after reconstitution showing bright-field [differential interference contrast (DIC)], Mag-green fluorescence (green), and Cy5-PE–labeled liposomal membrane (red). Mag-green fluorescence imaging depicts intraliposomal fluorescence intensity before and after MgCl_2_ addition (bottom row). Scale bars, 5 μm (top) and 2 μm (bottom). (**C** and **D**) Normalized mean trace of Mag-green fluorescence intensity in human ERMA reconstituted proteoliposomes after addition of MgCl_2_ (arrow) at various concentrations in the presence of (50 μM) Mg-ATP. Quantification of ERMA-mediated Mg^2+^ uptake in reconstituted proteoliposomes as Mag-green fluorescence following MgCl_2_ addition at increasing concentration. Data represent means ± SEM from *n* = 3 independent experiments. Mean ± SEM. ****P < 0.0001. (**E**) Titration curve of Mg^2+^ uptake following addition of various concentrations of MgCl_2_. Uptake increases with MgCl_2_ and saturates at ~5 mM. Mean ± SEM. (**F**) Normalized mean traces of Mag-Green fluorescence in proteoliposomes reconstituted with human ERMA (green), ERMA (D427N) mutant (red), or empty liposomes (blue) with the addition of 5 mM MgCl_2_ (arrow) in the presence of 50 μM Mg-ATP. Quantification of Mg^2+^ uptake as Mag-green fluorescence following the addition of 5 mM MgCl_2_. Data represent means ± SEM from *n* = 3 independent experiments (****P < 0.0001). f.a.u., fluorescence arbitrary units.

Since ERMA is an ER-localized 10-TM integral membrane protein, we next assessed whether ERMA functions as an ATP-dependent Mg^2+^ transporter under in vitro conditions, such as reconstitution of ERMA into proteoliposomes, and determined Mg^2+^ flux (Fig. 2A and fig. S3A). This assay directly determines its transport kinetics and coupling to ATP hydrolysis. We purified full-length human ERMA and an ERMA (D427N) mutant (fig. S3B), which were reconstituted into liposomes preloaded with the Mg^2+^ sensitive indicator Mag-green (Fig. 2A). This in vitro assay provides a direct assessment of ERMA’s Mg^2+^ transport activity. Bright-field and confocal fluorescence imaging demonstrated that the reconstitution procedure produced uniform vesicular proteoliposomes, with intact morphology and discrete luminal distributions of Mag-green and Cy5–phycoerythrin (PE) signals (Fig. 2A and fig. S3A). The proteoliposomes were suspended in ICM without Ca^2+^ (≤50 nM) (*44*, *45*) and exhibited markedly low Mag-green fluorescence before MgCl_2_ addition, indicating that vesicles were impermeable and therefore that subsequent fluorescence changes reflect bona fide transport rather than passive leakage. To assess the ERMA-mediated Mg^2+^ flux, proteoliposomes were incubated in Mg^2+^ free ICM buffer, and exogenous MgCl_2_ was pulsed at concentrations of 1, 2, and 5 mM in the presence of Mg-ATP (50 μM). ERMA-reconstituted proteoliposomes exhibited an increase in Mag-Green signal immediately after MgCl_2_ addition, followed by a plateau phase that was stably maintained for approximately 250 s. Purified microsomes from WT and *Erma^+/−^* hepatocytes revealed similar kinetics, indicating that this proteoliposome flux measurement recapitulates the physiological _ER_Mg^2+^ uptake mediated by ERMA (*24*). The persistence of the elevated signal suggested that liposomes remained structurally intact and retained the accumulated Mg^2+^ throughout the assay period (Fig. 2C). By contrast, empty liposomes exhibited nominal fluorescence changes under identical conditions, demonstrating that Mg^2+^ uptake was strictly dependent on ERMA incorporation into the membrane. Quantitative analysis of liposomes traces revealed a significant and concentration-dependent increase in ER luminal Mg^2+^ following MgCl_2_ addition (Fig. 2, C and D). At 1 mM MgCl_2_, a measurable rise in Mag-Green fluorescence was detected, whereas 2 and 5 mM concentrations produced a dose-dependent higher uptake (Fig. 2D). A concentration response curve showed that ERMA-mediated Mg^2+^ uptake followed saturating kinetics, with near-maximal accumulation reached at ~5 mM MgCl_2_ (Fig. 2D). These results demonstrate that the purified ERMA exerts ATP-dependent Mg^2+^ flux upon reconstitution into liposomes. To determine whether transport relies on the conserved aspartate phosphorylation, we introduced a mutation in the aspartate residue of the phosphorylation loop (DKQGIL motif), substituting Asp^427^ with Asn (D427N). This residue is likely required for the formation of a transient phosphorylated aspartyl-intermediate in P-type ATPases (*24*). Liposomes reconstituted with the ERMA (D427N) mutant exhibited no detectable Mg^2+^ accumulation following 5 mM MgCl_2_ addition, with fluorescence signals indistinguishable from those of protein-free liposomes (Fig. 2F). However, human ERMA reconstituted liposomes showed a marked increase in luminal Mg^2+^ under identical conditions (Fig. 2F). Collectively, these findings indicate that ERMA is an ATP-dependent Mg^2+^ transporter.

### Structural determination of human and mouse ERMA

**Fig. 3.**
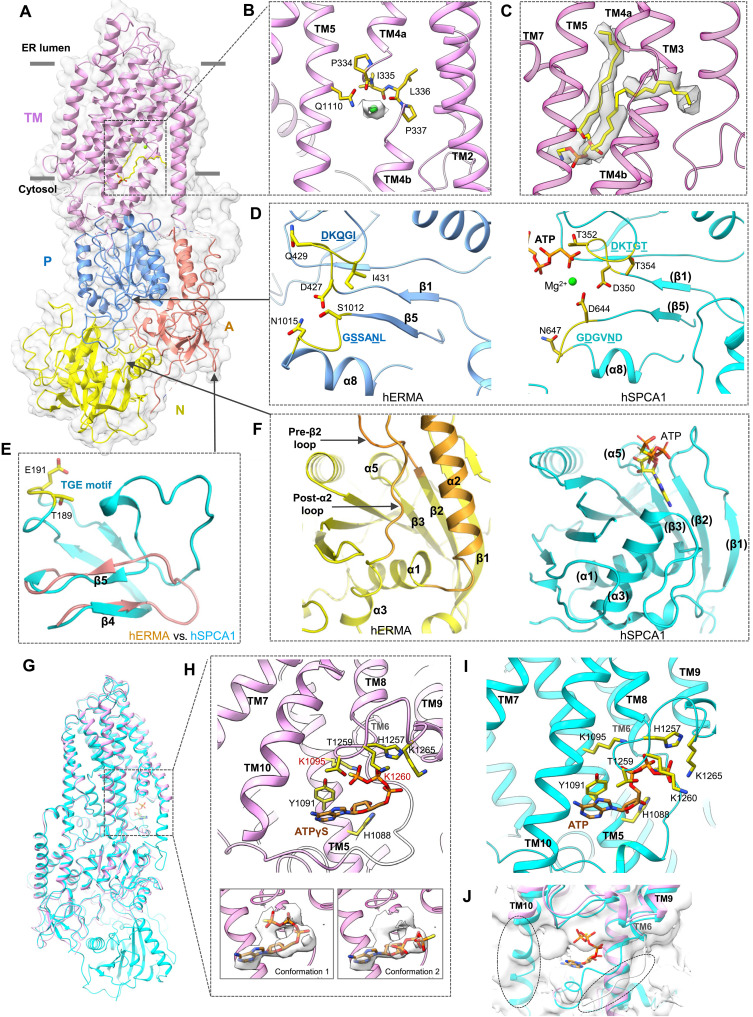
Structural studies of human and mouse ERMAs. (**A**) Overall structure of human ERMA (hERMA) with the TM and cytosolic domains individually colored. (**B**) Zoomed-in view of the ion-binding site in the unwound TM4 region. The density (gray surface) is modeled as Mg^2+^ (green sphere). (**C**) Zoomed-in view of the buried lipid (gray surface density modeled as POPE) within the TM domain of hERMA. (**D**) Comparison of hERMA (left) with a P-type ATPase (right; hSPCA1, PDB: 8IWR) (*75*) at the P-domain phosphorylation site. The highly conserved DKTGT and GDGVND motifs in P-type ATPase become DKQGI and GSSANL, respectively, in ERMA. Side chains of those underlined residues are shown in sticks. The secondary structure elements in hSPCA1 are labeled in brackets using the equivalent elements of hERMA. (**E**) Comparison of hERMA (salmon) and hSPCA1 (cyan) at the A-domain TGE motif. In hSPCA1, the long β4-β5 linker contains the TGE motif (yellow sticks) required for dephosphorylation in P-type ATPases, whereas the corresponding region in ERMA is a short loop lacking this motif. (**F**) Comparison of hERMA (left) and hSPCA1 (right) at the N-domain nucleotide-binding site. In hERMA, the nucleotide-binding pocket is occluded by α2, post-α2 loop, and pre-β2 loop (all colored in brown). (**G**) Overall structural comparison between mouse (purple) and human (cyan) ERMAs. (**H**) Zoomed-in view of the putative ATP-binding site in mouse Erma. Surrounding residues are shown as stick representation; two insets display the density (gray surface) for ATP-γ-s modeled in two conformations. (**I**) AlphaFold-predicted ATP-binding site in ERMA. (**J**) Structural comparison of mouse (purple) and human (cyan) ERMAs at the putative ATP-binding site. The post-TM6 loop and the C-terminal part of TM10 (both indicated in the ovals) became disordered in mouse ERMA upon ATP-γ-s binding.

To elucidate the structural basis of Mg^2+^ transport by ERMA, we purified detergent-solubilized human ERMA and determined its single-particle cryo-EM structures under three different conditions (see Materials and Methods): (i) in the absence of Mg^2+^ and nucleotide, (ii) in the presence of 5 mM Mg^2+^ and 1 mM Adenosine 5′-(β,γ-methylenetriphosphate) (AMPPCP), and (iii) in the presence of BeF_3_^−^ without Mg^2+^ (fig. S4 and table S1). All three reconstructions yielded nearly identical structures, and no bound BeF_3_^−^ or nucleotide was observed. The structure obtained in the presence of AMPPCP and Mg^2+^ exhibited the highest resolution [(2.7 Å (St. Jude Children’s Research Hospital) and 2.8 Å (The University of Texas Southwestern Medical Center)] and the best-defined soluble N domain (fig. S4, A to C) and is therefore used as the representative model in this study. Notably, a recent structural study of human ERMA also revealed identical conformations [Protein Data Bank (PDB): 9JJK, 9JJN, 9JJO, 9JK3, 9JK4, and 9JK5]. The overall architecture of ERMA resembles that of P-type ATPases, comprising 10-TM helices and three cytosolic domains corresponding to the A, P, and N domains (Fig. 3A). Among the three structures, the N domain was resolved only in the AMPPCP sample (fig. S4, A to C), suggesting its intrinsic flexibility. Similar to P-type ATPases, TM4 of ERMA breaks into two segments (TM4a and TM4b) by an unwound region in the middle with a sequence of 334PILP337 (Fig. 3B). In P-type ATPases, this unwound region harbors the conserved binding site for the transported ion. In ERMA, an ion-like density is located within this unwound region of TM4—analogous to the canonical metal-binding site of P-type ATPases and likely corresponds to a bound Mg^2+^ ion (Fig. 3B). The backbone carbonyls of P334 and L336 in the unwound TM4 region and the side chain of Q1110 in TM5 directly coordinate this ion, and mutation of Q1110 markedly reduces the ERMA Mg^2+^ uptake activity (see the “Domain-specific mutagenesis identifies catalytic and regulatory residues governing ERMA-dependent Mg^2+^ transport” section). Two elongated densities, likely corresponding to lipid acyl chains, were observed penetrating deeply into the cytosolic leaflet of the TM domain. One acyl chain is enclosed by TMs 3, 4b, and 5, while the other is surrounded by TMs 3 and 7 (Fig. 3C). A similarly positioned lipid was also reported in the recent ERMA structural study. It is unclear whether this deeply buried lipid merely stabilizes the protein or also plays a functional regulatory role (*46*). Several distinctive structural features of ERMA include the following: First, although ERMA contains a DKQGI sequence in its P domain that aligns with the conserved DKTGT phosphorylation motif of P-type ATPases (Fig. 3D and fig. S4D), the GDGVND loop—essential for Mg^2+^ coordination and the phosphorylation reaction in P-type ATPases—is replaced by GSSANL at the equivalent position in ERMA (Fig. 3D). Second, the TGES motif, which mediates dephosphorylation in P-type ATPases, is absent in ERMA (Fig. 3E). Third, the nucleotide-binding pocket that resides in the N domain of P-type ATPases is occluded in ERMA by additional structural elements, including a helix-loop (α2-loop) between α1 and α3, and an extended loop preceding β2 (Fig. 3F). However, we cannot exclude the possibility that conformational changes in alternative states may permit ATP access to the canonical nucleotide-binding pocket in ERMA. Notably, although the A, P, and N domains of ERMA can each be individually aligned with those of P-type ATPases, their relative orientations in ERMA differ markedly from any known conformations of P-type ATPases in various catalytic states, making it challenging to assign the current ERMA structure to a specific conformational state. To further probe potential ATP binding in ERMA, we also determined the cryo-EM structure of mouse ERMA in the presence of 5 mM adenosine 5′-[γ-thio] triphosphate (ATP-γ-S) (see Materials and Methods and fig. S4B). The overall structure closely resembles that of human ERMA, and its N domain remains poorly resolved (Fig. 3G). However, additional density was detected in the mouse *Erma* structure (Fig. 3H). The density is situated at the cytosolic membrane interface beneath the C terminus of TM8 and can be modeled as an ATP-γ-S molecule in two alternative conformations (Fig. 3H). A similar ATP-binding site was also predicted by AlphaFold (Fig. 3I). Among residues forming this putative ATP-binding pocket, mutations of K1101 and K1266 (equivalent to K1095 and K1260 in mouse ERMA) severely impair Mg^2+^ transport by human ERMA (see the “Domain-specific mutagenesis identifies catalytic and regulatory residues governing ERMA-dependent Mg^2+^ transport” section). Compared with the apo human ERMA structure, ATP-γ-S binding induces localized conformational rearrangements: The post-TM6 loop, which occludes the nucleotide site in the apo state, becomes disordered in the mouse structure, and the cytosolic portion of the unusually long TM10 helix also becomes disordered upon ATP-γ-S binding (Fig. 3J). Further investigations are required to verify whether this putative site represents the physiological ATP-binding site of ERMA and to elucidate the mechanism by which ATP binding and hydrolysis regulate its Mg^2+^ transport activity.

### ERMA catalysis depends on ATP and is modulated by ATP analogs and inhibitors

**Fig. 4.**
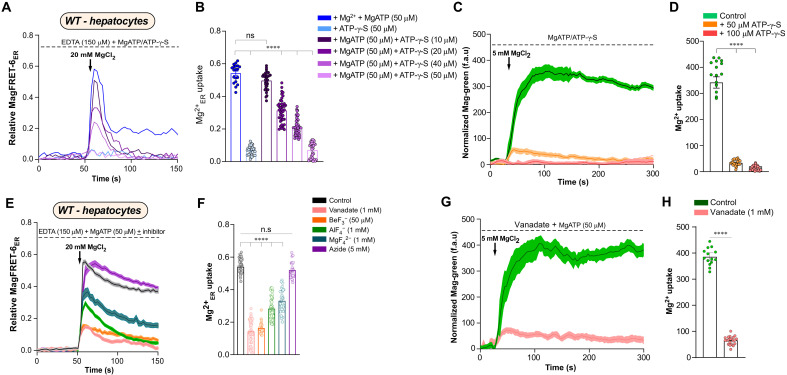
Modulation of ERMA activity by ATP analogs and P-type ATPase inhibitors. (**A** and **B**) WT murine hepatocytes were transfected with the MagFRET-6_ER_ sensor and maintained under physiological culture conditions to monitor ER luminal Mg^2+^ dynamics. After 72 hours, the cells were treated with increasing concentrations of ATP-γ-S in the presence or absence of Mg-ATP. Normalized mean traces of MagFRET-6_ER_ response following the addition of MgCl_2_. Quantification of _ER_Mg^2+^ uptake as MagFRET-6_ER_ response under the indicated conditions. Data represent the means from *n* = 3 independent experiments. (**P < 0.01; ****P < 0.0001). (**C** and **D**) Normalized mean trace of Mag-green fluorescence in human ERMA reconstituted proteoliposomes treated with 50 μM ATP-γ-S (orange) and 100 μM ATP-γ-S (red) following addition of 5 mM MgCl_2_ in the presence of 50 μM Mg-ATP. Quantification of _ER_Mg^2+^ uptake as Mag-green fluorescence under the indicated conditions. Data represent the means from *n* = 3 independent experiments. (n.s., not significant; ****P < 0.0001). (**E** and **F**) WT murine hepatocytes were transfected with the MagFRET-6_ER_ sensor and were treated with various inhibitors after 72 hours. Normalized mean trace of MagFRET-6_ER_ response following the addition of 20 mM MgCl_2_ in the presence of 50 μM Mg-ATP. Quantification of _ER_Mg^2+^ uptake as MagFRET-6_ER_ response under the indicated conditions. Data represent the means from *n* = 3 independent experiments. (n.s., not significant; ****P < 0.0001). (**G** and **H**) Normalized mean trace of Mag-green fluorescence in human ERMA reconstituted proteoliposomes treated with 1 mM vanadate (red) following addition of 5 mM MgCl_2_ in the presence of 50 μM Mg-ATP. Quantification of _ER_Mg^2+^ uptake as Mag-green fluorescence under the indicated conditions. Data represent the means from *n* = 3 to 5 independent experiments (****P < 0.0001).

To determine whether ERMA operates through the phosphorylation cycle of P-type ATPases, we performed an ATP-competition assay using the thiosulfate-containing nonhydrolyzable ATP, ATP-γ-S. We monitored ER luminal Mg^2+^ dynamics in digitonin-permeabilized murine hepatocytes expressing the MagFRET-6_ER_ sensor, revealing that 20 mM MgCl_2_ addition under control conditions resulted in robust _ER_Mg^2+^ uptake in the presence of 50 μM MgATP. However, supplementation of ATP-γ-S (10, 20, 40, or 50 μM) with Mg-ATP (50 μM) produced a dose-dependent inhibition of ERMA-mediated Mg^2+^ uptake (Fig. 4, A and B). This dose-dependent effect highlights the direct competition between Mg-ATP and ATP-γ-S at the nucleotide-binding site. Next, to assess whether the inhibitory effect directly affects ERMA activity, we reconstituted purified ERMA into Mag-Green encapsulated proteoliposomes to directly measure its transport function. Similarly, ATP-γ-S inhibited transport in a concentration-dependent manner (Fig. 4, C and D). These results demonstrate that ATP-γ-S sensitivity is an intrinsic property of ERMA. To further confirm whether ERMA activity is inhibited by a series of pan P-type ATPase inhibitors, ortho-vanadate, beryllium fluoride (BeF_3_^−^), aluminium fluoride (AlF_4_^−^), magnesium fluoride (MgF_4_^2−^), and sodium azide were tested in the permeabilized cell system. Upon addition of 20 mM MgCl_2_, we analyzed _ER_Mg^2+^ uptake in permeabilized murine hepatocytes treated with these inhibitors. Time-course traces of MagFRET-6_ER_ signal revealed that ortho-vanadate, BeF_3_^−^, AlF_4_^−^, and MgF_4_^2−^ strongly inhibited _ER_Mg^2+^ uptake compared to control. In contrast, sodium azide, an F-type ATPase inhibitor, had no significant effect on _ER_Mg^2+^ uptake (Fig. 4E). Quantification of MagFRET-6_ER_ signal confirmed that vanadate pretreatment exhibited near-complete inhibition of ERMA activity (Fig. 4F). In parallel, we examined the effect of vanadate in the proteoliposomes assay and found that ERMA-mediated Mg^2+^ uptake was fully inhibited (Fig. 4, G and H), consistent with a vanadate-sensitive transport mechanism.

### Domain-specific mutagenesis identifies catalytic and regulatory residues governing ERMA-dependent Mg^2+^ transport

**Fig. 5.**
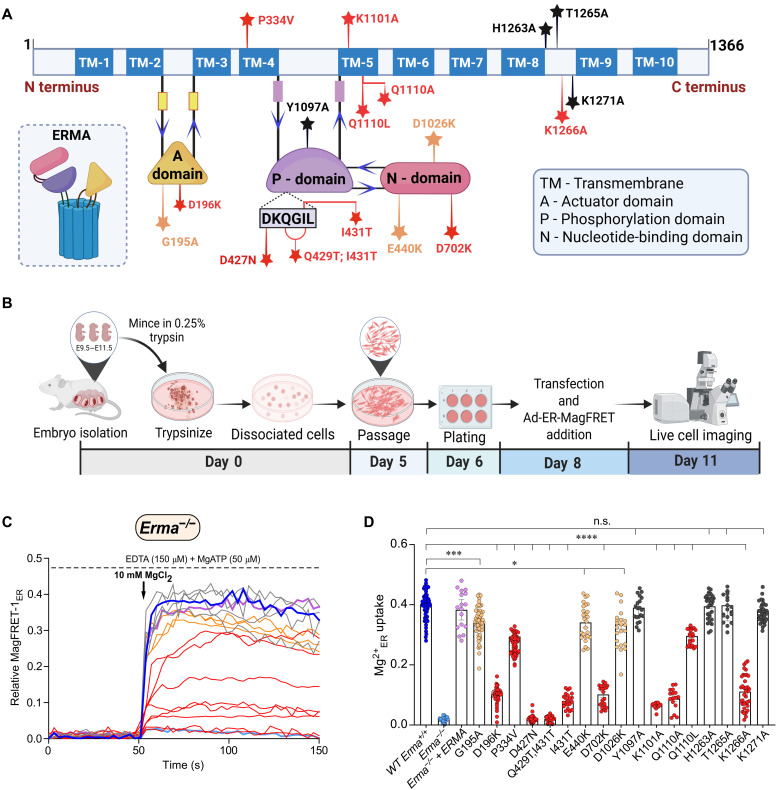
Mutational analysis reveals conserved residues essential for ERMA activity. (**A**) Schematic representation of ERMA protein topology highlighting the residues mutated and analyzed in this study. Created in BioRender. Venkatesan, M. (2026) https://BioRender.com/6jfw5qs. (**B**) MEFs Isolation and experimental workflow for the functional analysis of human ERMA functional mutants in murine MEFs. Embryos (E9.5 to E11.5) were isolated, and cells were transfected and transduced with MagFRET-1_ER_. Live-cell imaging was performed on day 11 to quantify _ER_Mg^2+^ uptake in the ERMA functional mutant constructs. Created in BioRender. Venkatesan, M. (2026) https://BioRender.com/dlabwk5. (**C**) WT and *Erma^−/−^* MEFs were transfected with the MagFRET-1_ER_ sensor. After 24 hours, *Erma^−/−^* MEFs were transfected with ERMA functional single/double mutants. Normalized mean traces of MagFRET-1_ER_ signal with the addition of 10 mM MgCl_2_ in the presence of 50 μM Mg-ATP and 150 μM EDTA. (**D**) Quantification of Mg^2+^ uptake as MagFRET-1_ER_ response under the indicated conditions. Data represent means ± SEM from *n* = 3 independent experiments and three to five replicates. Asterisks denote functional mutants in the ERMA protein (blue: WT *Erma^+/+^* murine hepatocytes; gray: no significant effect; orange: *P < 0.05; ***P < 0.001; red: ****P < 0.0001).

We next systematically assessed Mg^2+^ transport activity of a comprehensive panel of ERMA point mutants using permeabilized WT and *Erma^−/−^* mouse embryonic fibroblasts (MEFs) ectopically reconstituted with the MagFRET-1_ER_ sensor and the corresponding monomeric Red Fluorescent Protein (mRFP)-tagged human-ERMA mutant constructs. The selected mutations encompassed residues distributed across TM helices, cytosolic catalytic domains, and putative ion-coordination sites to interrogate their contributions to Mg^2+^ recognition and transport. Specifically, we analyzed G195A, D196K, P334V, D427N, Q429T + I431T, I431T, E440K, D702K, D1026K, Y1097A, K1101A, Q1110A, Q1110L, H1263A, T1265A, K1266A, and K1271A (Fig. 5A). Confocal fluorescence imaging in COS-7 cells confirmed precise ER targeting of all red fluorescent protein (RFP)–tagged mutant constructs coexpressed with MagFRET-1_ER_, as demonstrated by extensive colocalization within the reticular ER network (fig. S5). This systematic mutational approach enabled us to probe both conserved catalytic motifs and previously uncharacterized residues, thereby providing mechanistic insight into the structural determinants of ERMA-mediated Mg^2+^ uptake. Under conditions optimized for monitoring _ER_Mg^2+^ uptake (Fig. 5A), *Erma^−/−^* MEFs expressing the MagFRET-1_ER_ sensor were permeabilized and resuspended in ICM with 50 μM MgATP. Following the addition of 10 mM MgCl_2_, MEFs reconstituted with WT ERMA exhibited ATP-dependent Mg^2+^ uptake, consistent with a rapid ER luminal Mg^2+^ accumulation. In contrast, several mutants, including D196K, P334V, D247N, Q429T + I431T, I431T, D702K, K1101A, Q1110A, Q1110L, and K1266A displayed markedly reduced Mg^2+^ uptake when compared to WT (Fig. 5, B and C). These residues map to highly conserved motifs within the P domain, the N domain, and the TM ion-binding pocket, supporting their critical roles in Mg^2+^ coordination and catalytic cycling. Notably, substitutions at the conserved aspartates D196, D427, and D702, residues implicated in catalytic phosphorylation or charge compensation during ion binding, presented significantly reduced Mg^2+^ uptake, underscoring their essentiality for transport. Likewise, mutations within the putative P and N domains (Q429T, I431T, and D1026K) disrupted uptake, consistent with disruption of the ion-conduction pathway. Mutations at the C-terminal (H1263A, T1265A, and K1271A) retained near-WT transport activity, suggesting that this region may play a regulatory rather than catalytic role in ERMA function (Fig. 5, C and D). These observations raise the possibility that distal structural elements contribute to ERMA regulation or stability rather than forming the core transport machinery. Collectively, our findings delineate functionally essential residues in ERMA that govern Mg^2+^ uptake, thereby providing mechanistic insight into the molecular mechanism of this previously unidentified ER-resident P-type ATPase. These results also highlight structure-function relationships within ERMA, distinguishing residues that form the catalytic core from those that appear dispensable or regulatory.

### ERMA-SERCA chimeras reveal the TM regions selective for Mg^2+^

**Fig. 6.**
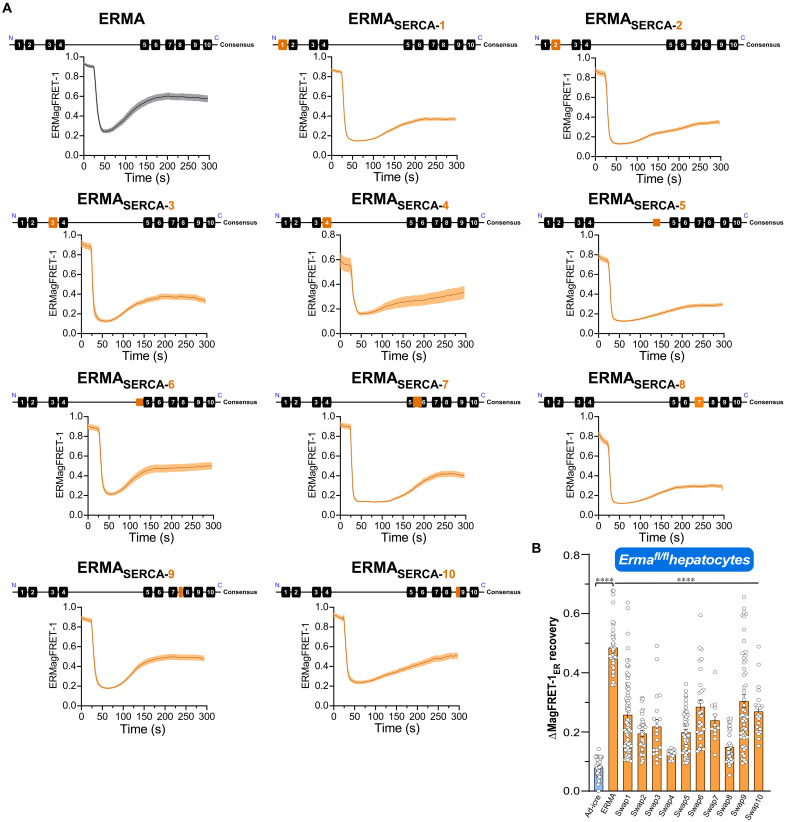
ERMA-SERCA chimeras reveal distinct TM determinants conferring Mg^2+^ and Ca^2+^ selectivity. (**A**) Representative MagFRET-1_ER_ mean traces from *Erma^fl/fl^* and Ad-iCre–treated hepatocytes expressing ERMA-SERCA1 chimeras signal after 5 mM lactate stimulation. In these constructs, individual TM helices of ERMA were replaced with the corresponding helices from SERCA1 (ERMA-SERCA-1 through ERMA-SERCA-10). (**B**) Quantification of MagFRET-1_ER_ responses across biological replicates. Data are expressed as means ± SEM. *N* = 3 independent experiments (****P < 0.0001).

A central question raised by our structural analysis of ERMA is which TM segments confer its unique specificity for Mg^2+^ transport compared to that of canonical Ca^2+^ P-type ATPases such as SERCA. Upon closer examination of the individual TM regions, we found that ERMA contains fewer acidic residues than SERCA. Having recognized the unique ion permeation mechanisms between Ca^2+^ and Mg^2+^, in particular, bacterial CorA or _m_Mg^2+^ uptake via MRS2 is gated by a GMN motif that is absent in MCU-like Ca^2+^ channels (*25*, *47*–*51*). Amino acid sequence alignment of ERMA across species revealed a conserved GMN motif positioned in the extreme C-terminal region (*24*). As the ERMA GMN motif is not positioned within either the TM helices or the cytosolic catalytic domains, it is likely to participate in regulatory processes rather than directly mediating ion permeation (*24*). To understand the ERMA-mediated Mg^2+^ permeation, we generated a panel of ERMA-SERCA1 chimeric constructs in which individual TM helices of ERMA were systematically replaced with the corresponding regions of human SERCA1a. Further, we evaluated SERCA1a structural compatibility with ERMA by generating ERMA-SERCA1a chimeric AlphaFold3-driven predictions (*52*). Regardless of swapping TM domains conserved between both ERMA and SERCA1a (TM1 to TM4), linking TM regions (TM5 and TM6), ER-luminal regions or cytosolic regions, the chimeric structures all showed higher mean overall predicted aligned errors (PAE) and higher swapped residue segment PAE scores relative to all other residues, indicating a general incompatibility of the SERCA1a sequences within ERMA (fig. S6, A to K). We generated four chimeras (fig. S6, A to K, D, F, G, and I; based on UniProt O14983 annotation) in which the SERCA Ca^2+^-binding residues were strategically swapped into the ERMA scaffold. Since the global *Erma* knockout mouse model is lethal, we generated an *Erma^fl/fl^* mouse model for acute or germline deletion (Fig. 1) (*24*, *41*). These human ERMA chimeric plasmid constructs were reconstituted in *Erma^fl/fl^ +* Ad-iCre hepatocytes, and then hepatocytes were transduced with MagFRET-1_ER_ sensor to assess _ER_Mg^2+^ uptake (fig. S6L). The _ER_Mg^2+^ uptake activity of these chimeras was then assessed using the MagFRET-1_ER_ sensor in primary hepatocytes. *Erma^fl/fl^ +* Ad-iCre hepatocytes were stimulated with l-lactate (5 mM) to trigger the release of _ER_Mg^2+^ and _ER_Mg^2+^ refilling (Fig. 6A and fig. S6M). Reconstitution of human ERMA WT in *Erma^fl/fl^ +* Ad-iCre hepatocytes restored _ER_Mg^2+^ uptake, which was absent in *Erma^fl/fl^ +* Ad-iCre hepatocytes (Fig. 6, A and B, and fig. S6M), confirming that ERMA is required for _ER_Mg^2+^ refilling. The sequential substitution of each SERCA TM helix with ERMA resulted in a marked loss of transport activity (Fig. 6, A and B). Notably, replacement of TM4 and TM8 exhibited near complete loss of _ER_Mg^2+^ (Fig. 6B). Despite the presence of these Ca^2+^ transport elements, all chimeras exhibited suppressed Mg^2+^ entry into the ER (Fig. 6), thus reinforcing a potentially distinct Mg^2+^ handling mechanism for ERMA. While the elevated PAE scores suggest that these segment swaps may introduce structure instability contributing to this attenuation, these findings also highlight a fundamental divergence in ion-binding architecture; consequently, future targeted point mutations of conserved TM4/TM8 residues will be essential to definitively decouple global structural incompatibilities from the specific residues governing Mg^2+^ transport and selectivity compared to Ca^2+^-selective SERCA1a (*53*, *54*).

### Human pathogenic ERMA mutations disrupt Mg^2+^ transport

**Fig. 7.**
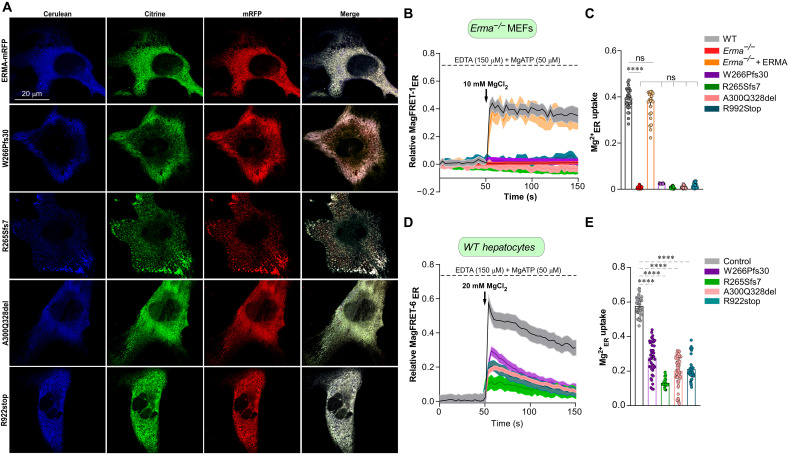
Pathogenic ERMA variants disrupt Mg^2+^ transport and define regions essential for activity. (**A**) Representative confocal images of *Erma^−/−^* MEFs coexpressing MagFRET-1_ER_ in WT ERMA (Control) or human pathogenic ERMA mutants mRFP constructs (W266Pfs30, R265Ss7, A300Q328del, and R922Stop). Scale bars, 20 μm. (**B** and **C**) WT and *Erma^−/−^* MEFs were cotransfected with the MagFRET-1_ER_ and ERMA human pathogenic mutants. Normalized mean traces of MagFRET-1_ER_ response following the addition of 10 mM MgCl_2_ in the presence of 50 μM Mg-ATP. Quantification of Mg^2+^ uptake as MagFRET-1_ER_ response under the indicated conditions. Data represent means ± SEM from *n* = 3 independent experiments. (n.s., not significant; ****P < 0.0001). (**D** and **E**) WT murine hepatocytes were cotransfected with the MagFRET-6_ER_ and human ERMA pathogenic mutants. Normalized mean traces of MagFRET-6_ER_ response following the addition of 20 mM MgCl_2_ in the presence of 50 μM Mg-ATP. Quantification of Mg^2+^ uptake as MagFRET-6_ER_ under the indicated conditions. Data represent means ± SEM from *n* = 3 independent experiments. (n.s., not significant; ****P < 0.0001).

Mutations in ERMA identified in humans have been linked to a multiorgan developmental disorder involving the brain, craniofacial structures, and both structural and developmental cardiac abnormalities, accompanied by distinct facial dysmorphism and neurodevelopmental impairment, underscoring the importance of its ion transport function in maintaining a steady state of [Mg^2+^]_ER_ (*24*, *41*, *55*). To understand this spectrum, we focused on four representative pathogenic alleles, W266Pfs30, R265Sfs7, A300Q328del, and R922stop, that encompass frameshift, truncation, and in-frame deletion variants reported in patients (*41*). These mutations map to distinct structural regions of ERMA, including the N-terminal TM core (W266Pfs30 and R265Sfs7), the cytosolic loop spanning TM4 and TM5 (A300Q328del), and the distal C-terminal domain (R922stop), thereby providing insight into how disruption of different structural elements compromises Mg^2+^ transport. Human pathogenic mutants were reconstituted in *Erma^−/−^* MEFs and WT primary hepatocytes (Fig. 7A and fig. S7, A and B), and _ER_Mg^2+^ uptake was quantified in permeabilized cells using the MagFRET-6_ER_ and MagFRET-1_ER_ sensors, respectively. Relative _ER_Mg^2+^ uptake was then quantified and compared across all conditions. WT ERMA exhibited robust ATP-dependent Mg^2+^ uptake, whereas multiple human mutants showed significant loss of function (Fig. 7, B to E). Overexpression of human ERMA disease–associated mutant constructs in WT hepatocytes markedly suppressed _ER_Mg^2+^ uptake, consistent with these variants conferring a loss-of-function phenotype. This link between defective ERMA activity and pathological phenotypes suggests that Mg^2+^ dysregulation in the ER may represent a previously underappreciated driver of organ development, metabolic, and neurological disease.

## DISCUSSION

Eukaryotic cells have evolved an exquisitely balanced network of ion transporters to coordinate both the rapid dynamics of signaling and the enduring demands of metabolism. ER has long been recognized for its role in Ca^2+^ storage and signaling, where flux through IP_3_ receptors, ryanodine receptors, and SERCA coordinates physiological and stress responses. Our findings extend this framework to Mg^2+^, demonstrating that the ER also serves as a dynamic Mg^2+^ reservoir, maintaining high luminal free Mg^2+^ and exhibiting regulated exchange during activation. Yet, the mechanisms maintaining the Mg^2+^ pool have remained poorly defined. The first observation of _ER_Mg^2+^ release and refilling was reported when primary hepatocytes were stimulated with glycolytic end product l-lactate (*25*). We subsequently identified that ERMA is responsible for _ER_Mg^2+^ refilling when l-lactate stimulation resulted in rapid _ER_Mg^2+^ depletion (*24*). With this knowledge, we used a permeabilized cell system where we could deplete the ER free Mg^2+^ passively by keeping a pan chelator EDTA in the extra ER milieu and quantify the ERMA-mediated Mg^2+^ uptake after a bolus of Mg^2+^ addition (*24*, *56*). This assay allowed us to quantitatively measure free [Mg^2+^]_ER_ using MagFRET_ER_ sensors with distinct *K*_ds_. The _ER_Mg^2+^ quantitative data were confirmed by comparing with an *Erma-*deficient condition (Fig. 1 and fig. S1). Our estimation of free [Mg^2+^]_ER_ was in the range between 15 and 30 mM, which is equivalent to the cytosolic bound Mg^2+^ concentration (*57**–**59*), conceptually establishing the ER as a bi-ionic reservoir.

This analysis revealed dose-dependent, physiologically relevant responses, enabling real-time tracking of _ER_Mg^2+^ flux. Our comprehensive compartmental and temporal examination is a substantial advancement over bulk assays such as inductively coupled plasma mass spectrometry or colorimetric assays. The understanding of ERMA function and structural analysis provides a mechanistic insight into how the ER maintains a luminal free Mg^2+^ pool as a reservoir. Reconstituting the purified human ERMA and D427N mutant into proteoliposomes establishes that ERMA mediates ATP-dependent Mg^2+^ uptake rather than a passive exchanger (Figs. 2 and 3). This mechanistic distinction is crucial as Mg^2+^ transporters identified thus far, including CorA, MgtE, and MRS2, typically rely on electrochemical gradients rather than ATP hydrolysis (*25*, *60*, *61*). Another unique feature of bacterial MgtA and its human ortholog ERMA is that Mg^2+^ not only stabilizes ATP as Mg-ATP but also simultaneously permeates into the ER lumen as the transported ion (*24*, *33*). This dual role establishes a self-referential transport loop that directly couples ATP-dependent catalysis to _ER_Mg^2+^ uptake. The context of regulatory mechanisms, Na^+^/K^+^ ATPase and SERCA activities are controlled by FXYD family proteins and phospholamban and sarcolipin β subunits. Similarly, a recent study revealed that ER-localized TUSC3 protein positively controls ERMA activity, suggesting the existence of a gatekeeping regulator of ERMA (*62*).

Our functional data indicate that the cryo-EM structure of human ERMA defines the tripartite cytosolic headpiece (A, P, and N domains) capping a compact multihelical TM core. At the catalytic core, ERMA’s P-domain loop centered on Asp^427^ adopts the geometric position of the aspartyl-phosphate acceptor, but the primary sequence motif is noncanonical (DKQGIL in place of DKTGTL). Functionally, D427N abolishes Mg^2+^ uptake in proteoliposomes, suggesting Asp^427^ as the catalytic site that couples nucleotide turnover to transport. However, three independent groups, including ours (two), have determined the overall architecture of human ERMA, and all available structural data reveal a nucleotide-binding pocket that is inaccessible to Mg-ATP or its analogs. Nonetheless, the present ERMA structure provides a framework to identify and define the key residues that participate in the _ER_Mg^2+^ uptake. Despite its overall architectural similarity to P-type ATPases, several distinct structural features of ERMA suggest that this _ER_Mg^2+^ transporter is distinct from the canonical P-type ATPase mechanism. Many key questions remain: Where is the physiological ATP-binding site located? How does ATP hydrolysis regulate ERMA-mediated Mg^2+^ transport? What conformational changes occur within the TM region and the cytosolic A, P, and N domains during the transport cycle? Does ERMA undergo phosphorylation-dephosphorylation transitions while mediating Mg^2+^ translocation? Resolving multiple structures of ERMA in distinct conformational states during Mg^2+^ transport will be essential to answer these outstanding puzzles.

Functional mutagenesis has been instrumental in elucidating the catalytic and ion selectivity and permeation of P-type ATPases, wherein conformational transitions are intimately coupled to phosphoryl transfer at a conserved aspartate residue within the DKTGT motif of the P domain. In ERMA, substitution of this residue (D427N) abrogated luminal Mg^2+^ uptake, underscoring its indispensability for catalytic phosphorylation. This mirrors the inactivating substitutions characterized in other P-type ATPases, such as SERCA (D351N), Na^+^/K^+^-ATPase (D369N), and H^+^/K^+^-ATPase (D386N), where the replacement of the catalytic Asp universally prevents the formation of the phosphorylated intermediate (E1 ~ P), thereby decoupling ATP hydrolysis from ion translocation. Building on the cryo-EM ERMA structure, systematic mutagenesis of ERMA uncovered additional structural and functional determinants governing _ER_Mg^2+^ uptake. Intriguingly, substitutions adjacent to the P-domain catalytic loop (Q429T + I431T, I431T) and within the N-domain region (D1026K, Y1097A, K1101A, Q1110A, and Q1110L) markedly reduced Mg^2+^ uptake, highlighting that the N-P domain coordination is likely essential to ion pumping. Likewise, mutations of acidic residues forming the predicted TM ion-binding pocket (D196K, P334V, and D702K) abolished Mg^2+^ transport, reinforcing the electrostatic integrity within the TM cavity that is tightly coupled to catalytic turnover (Fig. 5). Together, these results position ERMA as a mechanochemically conserved yet ion-selectivity divergent member of the P-type ATPase superfamily, where subtle variations in TM electrostatics and hydration govern Mg^2+^ specificity. Although all ERMA structures reported thus far by our group and others are highly similar, limiting mechanistic resolution at the current stage, our higher-resolution reconstruction provides several important advances. Specifically, it enabled confident identification of the Mg^2+^-coordinating site within the unwound TM4 region (Fig. 3), functional validation of key residues such as Q1110, and definition of a putative ATP-binding site at the cytosolic membrane interface supported by cryo-EM density, AlphaFold prediction, and loss-of-function mutagenesis. Moreover, although phosphorylation of the canonical aspartate could not be directly visualized, the loss of Mg^2+^ transport activity in the D427 mutant functionally supports a phosphorylation-dependent step in ERMA-mediated transport (Fig. 2).

To dissect the evolutionary and mechanochemical conservation among P-type ATPases, it is essential to recognize that the physicochemical diversity of their transported ions, from protons and Ca^2+^ to trace metals, demands precise tuning of TM electrostatics and hydration dynamics. The ER-resident Mg^2+^-ATPase ERMA provides a unique opportunity to examine how this scaffold adapts to transport the high charge density but smallest and highly hydrated divalent cation in biology. We systematically replaced individual ERMA TM helices (TM1 to TM10) with the corresponding helices from SERCA, a Ca^2+^-transporting P2A-ATPase, and measured Mg^2+^ uptake using MagFRET-1_ER_ sensor (Fig. 6). MagFRET-1_ER_–based _ER_Mg^2+^ uptake assays of the chimeric panel demonstrate a progressive decline in transport activity with increasing SERCA substitution, culminating in complete loss of Mg^2+^ uptake when TM helices were replaced. The resulting construct provides a direct experimental bridge linking ERMA’s divergent sequence and Mg^2+^ selectivity. The ERMA-SERCA chimeras reveal that Mg^2+^ selectivity arises not from the catalytic site but from the TM chemistry of a hydrated, polar, and conformationally flexible cavity, which is indispensable for stabilizing Mg^2+^ during ATP-driven translocation. Substitution of SERCA helices converts this environment into a dehydrating, Ca^2+^-optimized landscape, abolishing Mg^2+^ transport despite preserved catalytic potential. These results define a biophysical boundary between Mg^2+^ and Ca^2+^-pumping ATPases, illustrating how a conserved P-type framework can be evolutionarily retuned through subtle sequence remodeling to sequester Ca^2+^ and Mg^2+^ selectively. Furthermore, our data suggest that ERMA TM helices are selective for Mg^2+^ over Ca^2+^ because of the lack of SERCA-like isoforms and functional redundancy (*53*, *54*). This strategy provides a framework for future structural determination of selective chimeras and comparative evaluation of human pathogenic variants.

Human pathogenic ERMA variants associated with multisystem developmental syndromes underscore the essential role of _ER_Mg^2+^ transport in organogenesis and neurodevelopment. Functional assays demonstrated that frameshift (W266Pfs30 and R265Sfs7), in-frame deletion (A300Q328del), and truncation (R922stop) mutants cause a near-complete loss of ATP-dependent Mg^2+^ uptake in both WT hepatocytes and *Erma*^−/−^ MEFs (Fig. 7). Both W266Pfs30 and R265Sfs7 are localized to the N-terminal cytosolic region upstream of the membrane-embedded transport core. These frameshift mutations introduce early truncations that are predicted to eliminate the downstream TM helices required to form the Mg^2+^ permeation pathway, thereby preventing proper folding and transport function. The A300-Q328del variant corresponds to an in-frame deletion within the structurally resolved TM3 TM helix. Loss of this segment is predicted to disrupt local helical packing within the membrane core, impairing the conformational framework necessary for substrate access and gating-coupling transitions essential for Mg^2+^ translocation. The R922stop mutation truncates the protein upstream of the C-terminal TM bundle, removing critical helices required to form the ion conduction pathway and execute the transport cycle. Collectively, although arising from distinct lesion types, these variants are predicted to impair Mg^2+^ transport either by eliminating essential TM elements of the transport core (W266Pfs30, R265Sfs7, and R922stop) or by directly perturbing a structurally resolved TM helix required for proper helical packing and gating-coupling transitions (A300-Q328del). In comparison to other P-type ATPases such as SERCA and Na^+^/K^+^ ATPase, catalytic site substitutions (D351N or D369N) abolish phosphorylation and trap the enzyme in an E1-like conformation, resulting in congenital muscle and neurological phenotypes. Likewise, C-terminal truncations in ATP7A or ATP7B disrupt domain coupling and underlie Menkes and Wilson diseases. ERMA pathogenic variants converge on analogous mechanochemical failure modes, including loss of phosphorylation-dependent gating, collapse of the ion binding cavity, and disruption of interdomain coordination within a Mg^2+^ adapted electrostatic landscape. Together, these findings integrate functional, structural, and pathological insights, defining ERMA as a phosphorylation-gated Mg^2+^ pump whose pathogenic mutations are likely associated with _ER_Mg^2+^ deficiency, which leads to systemic developmental failure (*24*, *41*).

Although our structural analyses identify ERMA as a Mg^2+^ transporting P-type like ATPase, several important limitations remain. Future studies are required to define the Mg^2+^:ATP transport stoichiometry of ERMA and to determine whether transport is coupled to H^+^ countermovement. Such biochemical and biophysical analyses are essential to establish how ERMA compares mechanistically with other P-type ATPases and to clarify the energetic basis of _ER_Mg^2+^ uptake. The D427N mutation confirms the essential role for Mg^2+^ uptake, our data do not yet define how Mg^2+^ binding couples to ATP hydrolysis or whether ERMA undergoes the canonical E1-E2 transitions. Although the canonical TGES motif is indispensable for dephosphorylation in P-type ATPases, future studies are required to identify the corresponding residue in ERMA that fulfils an equivalent catalytic role. The lipid-reconstituted ERMA (nanodisc) structure is indistinguishable from the detergent-solubilized form, indicating overall conformational stability. Nevertheless, future studies will be required to delineate how the lipid bilayer environment influences ERMA’s domain organization, conformational transitions, and catalytic activity. Capturing substrate or nucleotide-bound intermediates at higher resolution, combined with phosphoenzyme trapping and proteomic mapping of interacting partners, will be critical to reconstruct its catalytic cycle and regulatory network. Together, these studies will clarify whether ERMA represents a divergent branch of the P-type ATPase superfamily and advance our understanding of _ER_Mg^2+^ dynamics and their role in protein-folding capacity, and organellar architecture.

In summary, the present results reveal that ER is a major intracellular compartment for free Mg^2+^ storage, as demonstrated by direct detection of ER luminal Mg^2+^ transport activity by ERMA and the loss of function by genetic deletion of Erma or reconstitution of pathogenic ERMA mutants. Our study shows that the lack of canonical catalytic TGES and DKTGT dephosphorylation/P domains are not the determinant of P-type ATPase architecture, but rather, the critical aspartate residue is necessary for ERMA activation. These unique features underscore the evolutionary flexibility of the P-type ATPase fold and lay the foundation for understanding how _ER_Mg^2+^ homeostasis supports protein folding, sterol biosynthesis, drug detoxification, and stress resilience.

## MATERIALS AND METHODS

### Cell lines and cell culture

COS-7 [American Type Culture Collection (ATCC) # CRL-1651] cells were grown in high-glucose Dulbecco’s modified Eagle’s medium (DMEM) (Hyclone, SH30022.01) supplemented with 10% (v/v) fetal bovine serum (FBS, Hyclone) and 1% (v/v) antibiotic-antimycotic. Cells were maintained at 37°C in a humidified incubator with 5% CO_2_. Transfections were performed using Lipofectamine 3000 (Thermo Fisher Scientific, L3000015). After 6 hours of transfection, the media were replaced with fresh growth media. After transfection, the cells were incubated for an additional 72 hours at 37°C, 5% CO_2_ before imaging.

### Plasmid constructs

All plasmid constructs used in this study were generated and sequence-verified by GenScript (Piscataway, NJ, USA). For ER-targeted sensors, constructs were designed with an N-terminal calreticulin signal sequence (SS) and an N-terminal KDEL ER-retention motif to achieve luminal localization. The following MagFRET_ER_ constructs were used: Calreticulin SS_MagFRET1, −5, −6, and 8. To investigate the structural and functional determinants of ERMA, WT and site-directed mutant constructs were generated in the pCMV6-AC mammalian expression backbone with either RFP or 3× FLAG C-terminal tags. The following point mutants were tagged with (G195A, D196K, P334V, D427N, Q429T, I431T, E440K, D702K, D1026K, Y1097A, K1101A, Q1110A, Q1110L, H1263A, T1265A, K1266A, and K1271A)–RFP-pCMV6-AC. Each mutant was sequence-verified and expressed at comparable levels to the WT construct (ERMA-RFP-pCMV6-AC). Human pathogenic variants corresponding to ERMA isoform-2 were also cloned into the pCMV6-RFP backbone, including A300Q328del, R265Sfs7, R922stop, and W266Pfs30, to model clinically observed loss-of-function mutations. In addition, a SERCA1-swap chimera [SERCA1-Swap(1-10)–ERMA-WT-RFP-pCMV6-AC] was generated to assess TM domain compatibility and potential mechanistic parallels between ERMA and P-type ATPase family members. All plasmids were verified by full sequencing, and DNA purity was confirmed via A260/A280 absorbance ratio and agarose gel electrophoresis before transfection.

### Animals

All mice were maintained in the institutional animal facility in accordance with protocols approved by the Institutional Animal Care and Use Committee of the University of Texas Health Science Center San Antonio (protocol number IPROTO202500000041). Both male and female mice were used between the ages of 12 and 16 weeks. Animals were bred with WT C57BL6/J mice to generate an initial cohort of *Erma*^+/−^, *Erma^fl/fl^*, and WT mice (*25*).

### Generation, genotyping, and conditional deletion of *Erma^fl/fl^* allele

The *Tmem94* (*Ermatm1a*)–targeted allele was obtained from the European Conditional Mouse Mutagenesis program. The *tm1a* allele contains a promotor-driven *lacZ-neo* reporter-selection cassette flanked by *FRT* sites and *loxP* sites flanking exon 6 of the *Erma* gene. To generate a conditional-ready allele, *Erma^tm1a/+^* mice were bred with ACTB-*Flp* deleter mice to excise the *FRT*-flanked cassette, producing the *Erma^tm1a(flox)^* allele. Heterozygous *Erma^tm1a^* mice were intercrossed to produce a homozygous *Erma^fl/fl^* colony. All mice were maintained on a *C57BL/6J* background, and both male and female mice aged 8 to 12 weeks were used for experiments. Genomic DNA was extracted from tail samples or hepatocytes using a standard alkaline lysis method. PCR-based genotyping was performed using primer sets specific for (i) the *loxP-*flanked *exon 6*, (ii) the *FRT*-junction confirming *Flp*-mediated cassette removal, and (iii) the Δ*exon6* recombined allele following *Cre* expression. PCR products were resolved on 1.5% agarose gels and compared with known genotype controls. For hepatocytes specific deletion, *Erma^fl/fl^* primary hepatocytes were isolated and transduced with adenoviral-iCre (*Ad-iCre*) at a multiplicity of infection of 20 (MOI-20) for 72 hours to allow efficient recombination and deletion of *Erma* expression, resulting in Cre-mediated excision of exon 6 and generation of the *Erma* conditional knockout hepatocytes.

### Isolation and culture of primary murine hepatocytes

Primary murine hepatocytes were isolated from 8- to 12-week-old male and female mice (WT, or *Erma^fl/fl^*) using a two-step collagenase perfusion method. Following cannulation of the portal vein, the liver was first perfused with Ca^2+^/Mg^2+^ free Hanks’ balanced salt solution buffer (35 mM Hepes and 0.75 mM EGTA) to remove blood and chelate divalent cations. In the subsequent step, perfusion with digestion medium (DMEM, Gibco, #12320) freshly supplemented with collagenase D (Worthington) was performed to enzymatically dissociate the extracellular matrix. After perfusion, liver lobes were excised, gently disrupted in isolation medium (DMEM supplemented with 10% FBS), and filtered through a 100-μm strainer to remove debris. The resulting cell suspension was subjected to three sequential centrifugation wash steps (50*g*, 5 min, 4°C), with each pellet resuspended in fresh isolation media. The cells were finally resuspended in Williams E medium (Sigma-Aldrich, #W4128) supplemented with 10% FBS (Hyclone), 1% antibiotic-antimycotic solution (Gibco), 2 mM l-glutamine, and 1% minimum essential medium nonessential amino acids (Gibco) and plated on collagen-coated culture dishes (Corning BioCoat). For imaging experiments, hepatocytes were seeded on in-house collagen-coated 25-mm glass coverslips. After 6 hours, attachment was confirmed, and the media were replaced with fresh warm Williams E medium. Cultures were maintained at 37°C in a humidified 5% CO_2_ atmosphere until use (*25*, *29*).

### MEFs isolation

*Erma^+/−^* pregnant mice at embryonic day (E9. to E11.5) were used for MEF isolation (*24*). After the heads, tails, limbs, and most internal organs were removed, the whole embryos were minced and digested in trypsin for 20 min at 37°C with gentle agitation and then seeded into 10-cm dishes with complete MEF media. MEFs were passaged two to three times to obtain a morphologically homogenous population and then expanded for experimental studies (split ratio 1:2). The genotyping of Erma embryos and mRNA abundance were described below. For studies using MagFRET-1_ER_ adenovirus, the cells were infected after 2 to 3 days in culture (MOI-10). For coexpression, MEFs were transfected with ERMA WT or its mutant constructs followed by adenoviral infection for 72 hours before confocal live imaging (*24*).

### Calibration of ER-targeted MagFRET sensors

Primary murine hepatocytes were isolated from WT mice and seeded onto 25-mm collagen-coated glass coverslips. After overnight culture, the cells were transiently transfected with 1 μg of organelle-targeted MagFRET plasmids using Lipofectamine 3000 (Thermo Fisher Scientific). For ER measurements, MagFRET constructs fused to calreticulin ensured ER localization and spanned a broad affinity range: MagFRET-1_ER_ (*K*_d_ ~ 0.15 mM), MagFRET-8_ER_ (*K*_d_ ~ 0.89 mM), MagFRET-5_ER_ (*K*_d_ ~ 7.4 mM), and MagFRET-6_ER_ (*K*_d_ ~ 15 mM). For _ER_Mg^2+^ uptake, hepatocytes were permeabilized in ICM [in milimolar, 120 KCl, 10 NaCl, 1 KH_2_PO_4_, and 20 Hepes-tris (pH 7)] containing digitonin (40 μg/ml, 2 min at 37°C), washed, and resuspended in fresh ICM lacking digitonin for live imaging. To measure _ER_Mg^2+^ uptake, permeabilized cells were bathed with ICM containing 150 μM EDTA, which triggered a rapid reduction of the MagFRET_ER_ signal, reaching a stable baseline ~200 s. Subsequent supplementation with 50 μM Mg-ATP followed by the addition of extracellular 10 mM MgCl_2_ showed a measurable increase in the FRET signal, reflecting ER luminal Mg^2+^ uptake. A bolus of indicated concentrations of MgCl_2_ were applied. Confocal FRET imaging was performed at 37°C on a Leica TCS SP8 equipped with a 63× oil immersion objective and FRET-SE detection module. Sequential line-by-line acquisition was used. After a 30-s baseline, Mg^2+^ was acutely depleted using ICM containing 150 μM EDTA (plus 50 μM Mg-ATP for ER assays), followed by stepwise additions of MgCl_2_ to generate in situ calibration curves. Once low baseline signals were reached, stepwise additions of MgCl_2_ (0.15 to 30 mM) were applied to establish calibration curves. FRET ratios were extracted using Leica FRET-SE software optimized for live-cell quantitative FRET analysis. We set the laser/detectors for the cerulean (donor) at 458-nm excitation, and the emission signal was collected at 460 to 490 nm for cross-talk correction. For the acceptor, the citrine was excited at 488 nm, and the emission signal was collected at 510 to 550 nm for cross-talk correction. For the FRET signal, cerulean was excited at 458 nm, and citrine (acceptor) and the FRET signal were collected at 510 to 550 nm. The following parameters were used for FRET-SE calculation. The FRET channel is denoted as B, and its signal is derived from the acceptor channel. Here, β refers to a donor cross-talk; β = B/A, which generates donor only reference correction factor. γ refers to and corrects for acceptor cross-excitation; γ = β/C. After these corrections, we calculated apparent FRET efficiency (*E*_A_) by using the following equation: *E*_A_ = β-A 3 × β – C × γ/C. FRET data were normalized to basal signals to compare the response between groups (*24*, *25*, *63*).

### Immunoblotting

Cell extracts were prepared from *Erma^fl/fl^* and Ad-iCre–transduced *Erma^fl/fl^* hepatocytes. Cells were transiently transfected with plasmids encoding ERMA-RFP or the indicated ERMA mutant RFP fusion constructs (W266Pfs30-RFP, R265Sfs7-RFP, A300Q328del-RFP, and R922Stop-RFP). The cells were collected 72 hours postinfection and lysed in receptor-interacting protein kinase buffer supplemented with protease and phosphatase inhibitors. Total protein concentration was determined using the Pierce BCA Protein Assay Kit (Thermo Fisher Scientific). Protein samples were prepared by heating at 90°C for 5 min in 4× LDS sample buffer with reducing agent. Equal amounts of protein were separated on 4 to 12% bis-tris polyacrylamide gels (Thermo Fisher Scientific) and transferred to polyvinylidene difluoride membranes. Membranes were blocked with 5% nonfat dry milk in Tris-Buffered Saline with Tween 20 (TBST) for 1 hour at room temperature, washed, and probed with the indicated primary antibodies. The polyclonal rabbit anti-ERMA antibody was custom-generated (1:500; YenZym Antibodies). Additional primary antibodies used include anti-SERCA2A (Invitrogen; 1:4000), anti-mRFP (Thermo Fisher Scientific, 1:2000), and anti–β-actin (Santa Cruz Biotechnology; 1:10,000). Horseradish peroxidase–conjugated secondary antibodies were obtained from Cytiva/Amersham. After antibody incubation and washing, bands were visualized using enhanced chemiluminescence and detected on autoradiography film.

### Preparation of proteoliposomes for ERMA reconstitution

*Escherichia coli* polar lipids and egg-yolk phosphatidylcholine (Avanti Polar Lipids) were mixed at a 3:1 ratio (w/w), each dissolved in chloroform at 25 mg/ml. The solvent was evaporated under a gentle nitrogen stream with continuous rotation to form a homogeneous lipid film, followed by overnight desiccation under vacuum. The dried film was hydrated in ICM buffer [20 mM Hepes and 150 mM KCl (pH 7.4)] containing 10 μM Mag-Green (Invitrogen, M3735) to a final lipid concentration of 25 mg/ml. Purified WT ERMA or D427N mutant was added to the hydrated liposomes at a protein to lipid ratio of 1:20 (w/w) and incubated for 1 hour at 4°C. To facilitate protein incorporation, preformed liposomes were briefly sonicated (1 to 3 s on ice) to transiently loosen the bilayer before addition of detergent solubilized ERMA, followed by five freeze-thaw cycles (liquid nitrogen and 40°C water bath) to promote membrane insertion and resealing. The resulting suspension was extruded 15 times through polycarbonate filters (Avanti) using a mini-extruder to generate large unilamellar vesicles (*44*). Cy5-PE (Avanti) was incorporated at 0.2 mol % of total lipid to label membranes. Control liposomes were prepared in parallel without the addition of protein. External Mag-Green was removed by ultracentrifugation. Proteoliposomes were diluted in ICM buffer and pelleted at 50,000*g* for 1 hour at 4°C, and the supernatant containing unencapsulated dye was carefully discarded. Pellets were gently resuspended in fresh ICM buffer, and this wash procedure was repeated three times to ensure complete removal of free dye. For image-based Mg^2+^ uptake assays, proteoliposomes were diluted into Mg^2+^ free ICM (with or without Mg-ATP) and imaged by a Leica SP8 confocal microscope using a 63× oil-immersion objective at room temperature. Luminal Mag-Green fluorescence was recorded before and after the addition of MgCl_2_ (1 to 5 mM), and intensity changes were quantified using Leica LAS X software (*2*).

### Protein expression and purification

For cryo-EM studies, full-length WT human ERMA [National Center for Biotechnology Information (NCBI) accession no. NP_001308077.1) or mouse ERMA (NCBI accession no. NP_082290.2) containing an N-terminal FLAG-tag (DYKDDDDK) was cloned into a pEZT-BM plasmid (*64*). *E. coli* DH10Bac cells were used to synthesize the bacmids that were used in baculovirus production in Sf9 cells (Thermo Fisher Scientific, 11496015) using Cellfectin II reagent (Thermo Fisher Scientific). For protein expression, human embryonic kidney 293S GnTI^−^ cells (ATCC, CRL-3022) grown in suspension at 37°C to a density of 3 × 10^6^ cells/ml were infected with P3 viruses at a ratio of 1:40 (virus: cell, v/v) and supplemented with 10 mM sodium butyrate to boost protein expression. The cells were then incubated at 37°C for 48 hours before being harvested by centrifugation (5000*g*, 15 min, 4°C). For the protein to be reconstituted into proteoliposomes, full-length WT and D427N mutant ERMA containing a C-terminal FLAG-tag (DYKDDDDK) were cloned into pEG BacMam plasmids. *E. coli* EMBacY cells were used to synthesize the bacmids that were used in baculovirus production in Sf9 cells (Thermo Fisher Scientific, 11496015) using X-tremeGENE transfection reagent (Roche). For protein expression, Expi293S GnTI^−^ cells grown in suspension at 37°C to a density of 3 × 10^6^ cells/ml were infected with P3 viruses at a ratio of 1:10 (virus:cell, v/v), The cells were then incubated at 37°C for 16 hours before supplementation with 10 mM sodium butyrate and incubated at 30°C for and additional 48 hours before being harvested by centrifugation. For cryo-EM studies, apo human ERMA purification, the cell pellet was resuspended in lysis buffer [50 mM tris-HCl (pH 7.5), 100 mM KCl, 5 mM β-Mercaptoethanol (β-ME), and 10% glycerol] supplemented with protease inhibitors [pepstatin (0.5 μg/ml), leupeptin (2 μg/ml), aprotinin (1 μg/ml), and 1 mM PMSF] and homogenized by sonication. ERMA was then extracted with 1% (w/v) *n*-dodecyl-β-d-maltopyranoside (Anatrace) supplemented with 0.2% (w:v) cholesteryl hemisuccinate (Sigma-Aldrich) in gentle agitation for 2 hours 4°C. The supernatant was subsequently collected by centrifugation (40,000*g*, 30 min, 4°C) and incubated with anti-DYKDDDDK G1 affinity resin (GenScript) for 1.5 hours at 4°C in gentle agitation. The resin was then washed with buffer A [50 mM tris-HCl (pH 7.5), 100 mM KCl, 5 mM β-ME, 10% glycerol, and 0.01% LMNG (lauryl maltose neopentyl glycol)], and elution was performed by incubating for 45 min at room temperature in gentle agitation with buffer B [50 mM tris-HCl (pH 7.5), 100 mM KCl, 5 mM β-ME, 10% glycerol, 0.01% LMNG, and FLAG peptide (0.2 mg/ml)]. The eluate was then concentrated and further purified by size exclusion chromatography in buffer C [20 mM tris-HCl (pH 7.5), 100 mM KCl, 1 mM dithiothreitol (DTT), and 0.007% LMNG] on a Superdex 200 Increase 10/300 GL (GE Healthcare). The purification procedure was the same for all other samples, except that: (i) In the Mg^2+^ and AMPPCP-containing human ERMA, all purification solutions contained additional 5 mM MgCl_2_, and 1 mM AMPPCP was added to the protein sample the night before grid preparation. (ii) For BeF_3_^−^-containing human ERMA without Mg^2+^, all solutions were treated with Chelex-100 resin before use; the purification solutions contained additional 10 mM NaCl and 1 mM KH_2_ PO_4_; buffer C contained additional 1 mM EDTA; the purified ERMA was treated with 5 mM BeSO_4_, 10 mM NaF, and 1 mM EDTA overnight before grid preparation. (iii) In the Mg^2+^ and ATP-γ-S–containing mouse ERMA, all purification solutions contained additional 5 mM MgCl_2_ and 1 mM CaCl_2_, and 5 mM ATP-γ-S was added to the protein sample the night before grid preparation. For the protein to be reconstituted into proteoliposomes, WT human ERMA cell pellets of WT and D427N ERMA were resuspended in lysis buffer [50 mM tris-HCl (pH 8.0), 300 mM KCl, and 1 mM DTT] supplemented with protease inhibitors. ERMA was then extracted with 1.5% (w/v) 2,2-didecylpropane-1,3-bis-β-d-maltopyranoside (LMNG, Anatrace) in gentle agitation for 1.5 hours at 4°C. The supernatant was collected by centrifugation (70,000*g*, 30 min, 4°C) and incubated with anti-Flag M2 affinity resin (Sigma-Aldrich) for 0.5 hours at 4°C in gentle agitation. The resin was then washed with buffer [50 mM tris-HCl (pH 8.0), 150 mM KCl, 1 mM DTT, 0.01% LMNG, 10 mM ATP, and 10 mM MgCl_2_], and elution was performed by three batch incubations with Flag peptide (0.2 mg/ml). The eluate was then concentrated and further purified by size exclusion chromatography in [25 mM tris-HCl (pH 8.0), 150 mM KCl, 1 mM DTT, and 0.005% LMNG] on a Superose 6 Increase 10/300 GL (GE Healthcare).

### Cryo-EM sample preparation and data acquisition

ERMA proteins were concentrated to ~0.5 mg/ml for grid preparation. For Mg^2+^ and AMPPCP containing human ERMA, 1 mM AMPPCP was added to the concentrated protein, and the sample was incubated overnight at 4°C before grid preparation. For BeF_3_^−^-containing human ERMA without Mg^2+^, 5 mM BeSO_4_, 10 mM NaF, and 1 mM EDTA were added to the concentrated protein, and the sample was incubated overnight at 4°C before grid preparation. For the Mg^2+^ and ATP-γ-S–containing mouse ERMA, 5 mM ATP-γ-S was added to the concentrated protein, and the sample was incubated overnight at 4°C before grid preparation. For grid preparation, 3.5 μl of the sample was applied to a glow-discharged Quantifoil R1.2/1.3 300-mesh gold holey carbon grid (Quantifoil, Micro Tools GmbH, Germany), blotted for 3.5 s with a blot force of 15 under 100% humidity at 12°C, and plunged into liquid ethane using a Mark IV Vitrobot (FEI). For the grid preparation of the WT human ERMA later used in reconstitution studies, the protein was concentrated to ~2.2 mg/ml and supplemented with 5 mM Mg^2+^ and 5 mM AMPPCP. For grid preparation, 3.5 μl of the sample was applied to a glow-discharged UltraAuFoil R1.2/1.3 300-mesh gold holey carbon grid (Quantifoil, Micro Tools GmbH, Germany), blotted for 3.0 s with a blot force of −2 under 100% humidity at 12°C, and plunged into liquid ethane using a Mark IV Vitrobot (FEI). For the dataset of apo human ERMA, micrographs were acquired on a Titan Krios microscope (FEI) operated at 300 kV with a Falcon4 electron detector (Thermo Fisher Scientific), using a slit width of 20 eV on a post-column Selectris X energy filter (Thermo Fisher Scientific). Data were collected with SerialEM using a Falcon4 camera with a pixel size of 0.738 Å. The defocus range was set from −0.9 to −2.2 μm. Each movie was dose-fractionated to 60 frames with a dose rate of 1e^−^/Å^2^/frame for a total dose of 60e^−^/Å^2^. The total exposure time was between 3.5 to 4 s. For the dataset of Mg^2+^ and AMPPCP containing human ERMA, micrographs were acquired on a Titan Krios microscope (FEI) operated at 300 kV with a K3 Summit direct electron detector (Gatan), using a slit width of 20 eV on a GIF-Quantum energy filter. Data were collected with SerialEM using the CDS (correlated double sampling) mode of the K3 camera with a super-resolution pixel size of 0.4135 Å. The defocus range was set from −0.9 to −2.2 μm. Each movie was dose-fractionated to 60 frames with a dose rate of 1e^−^/Å^2^/frame for a total dose of 60e^−^/Å^2^. The total exposure time was between 5 to 6 s. For the dataset of BeF_3_^−^-containing human ERMA, micrographs were acquired on a Titan Krios microscope (FEI) operated at 300 kV with a K3 Summit direct electron detector (Gatan), using a slit width of 20 eV on a GIF-Quantum energy filter. Data were collected with SerialEM using the CDS mode of the K3 camera with a super-resolution pixel size of 0.664 Å. The defocus range was set from −0.9 to −2.2 μm. Each movie was dose-fractionated to 60 frames with a dose rate of 1e^−^/Å^2^/frame for a total dose of 60e^−^/Å^2^. The total exposure time was between 3 and 4 s. For the dataset of Mg^2+^ and ATP-γ-S–containing mouse ERMA, micrographs were acquired on a Titan Krios microscope (FEI) operated at 300 kV with a K3 Summit direct electron detector (Gatan), using a slit width of 20 eV on a GIF-Quantum energy filter. Data were collected with SerialEM using the CDS mode of the K3 camera with a super-resolution pixel size of 0.51 Å. The defocus range was set from −0.9 to −2.2 μm. Each movie was dose-fractionated to 60 frames with a dose rate of 1e^−^/Å^2^/frame for a total dose of 60e^−^/Å^2^. The total exposure time was between 4 and 5 s. For the dataset of the WT human ERMA protein later used in reconstitution studies, micrographs were acquired on a Titan Krios microscope (FEI) operated at 300 kV with a K3 Summit direct electron detector (Gatan), using a slit width of 10 eV on a GIF-Quantum energy filter. Data were collected with EPU software in super-resolution mode with a super-resolution pixel size of 0.324 Å. The defocus range was set from −0.5 to −2.5 μm. Each movie was dose-fractionated to 60 frames with a dose rate of 1e^−^/Å^2^/frame for a total dose of 63.8 e^−^/Å^2^. The total exposure time was 3 s. The subframes were two times binned before motion correction.

### Cryo-EM data processing

Data processing was performed using cryoSPARC (*65*) following the general scheme described below. Movies were subjected to patch motion correction and subsequent patch CTF estimation. The resulting micrographs were curated to remove images with bad defocus values, ice contamination, and carbon. An initial round of particle picking was carried out with a blob picker. Particles were then extracted and subjected to one round of two-dimensional (2D) classification. Classes displaying clear features of the P-type ATPase were selected and used to repick particles with template or topaz picker. Additional rounds of 2D classification were further performed and particles from selected classes were used to obtain an initial 3D reconstruction with ab initio. Several rounds of 3D heterogeneous refinement were then used to remove junk particles. The resulting particles were subjected to 3D classification without alignment to differentiate channel conformations. The best-resolving 3D classes were reextracted with the original pixel size and refined using nonuniform refinement (*66*). During the refinement, defocus refinement with optimized per-particle defocus and global CTF refinement with optimization of per-group CTF parameters were enabled. Map resolutions were reported according to the gold-standard Fourier shell correlation using the 0.143 criterion (*67*). Local resolutions and angular distributions were estimated in cryoSPARC.

### Model building

Initial models were obtained using ModelAngelo (*68*). The models were then manually adjusted in Coot (*69*) and refined against the respective maps in Phenix (*70*). The geometry statistics of the models were obtained using MolProbity (*71*). The outliers were fixed using ISODE (*72*). All the structural figures were prepared using UCSF ChimeraX (*73*, *74*) and PyMOL (Schrödinger LLC., http://pymol.org).

### ERMA functional and pathogenic ERMA mutant screening

Primary murine hepatocytes and MEFs (WT and *Erma^−/−^*) were isolated and cultured on 25-mm collagen-coated glass coverslips. After overnight culture, cells were transiently transfected with 1 μg of plasmids encoding either ERMA functional mutants (G195A, D196K, P334V, D427N, Q429T and I431T, I431T, E440K, D702K, D1026K, Y1097A, K1101A, Q1110A, Q1110L, H1263A, T1265A, and K1271A) or human ERMA pathogenic mutants (truncation/frameshift/deletion) variants (R265Sfs7, R922stop, A300Q328del, and W266Pfs30) using Lipofectamine 3000 (Thermo Fisher Scientific). After 24 hours posttransfection, the cells were transduced with MagFRET-6_ER,_ and after 72 hour, hepatocytes/MEFs were permeabilized in ICM containing digitonin (40 μg/ml) for 2 min at room temperature, washed, and resuspended in 1 ml of fresh ICM without digitonin in the confocal chamber. Live-cell FRET imaging was performed at 37°C using a Leica TCS SP8 confocal microscope equipped with a 63× oil immersion objective and FRET-SE module. Following a 30-s baseline recording, the cells were perfused with ICM containing 150 μM EDTA to deplete ER luminal Mg^2+^. Once a stable low MagFRET signal was reached, 50 μM Mg-ATP was added followed by additions of MgCl_2_. Data acquisition and analysis were performed using the Leica FRET-SE module (SP8 system), which quantifies FRET efficiency in live cells.

### Measurement of ER Mg^2+^ uptake using MagFRET-6_ER_ under ATP analogs and inhibitor treatments

Primary murine hepatocytes were isolated and plated on collagen-coated, glass-bottom dishes, followed by transfection with MagFRET-6_ER_ sensor. Cells were maintained in standard hepatocyte culture medium and imaged after 72 hours of incubation. Immediately before imaging, the cells were incubated for 30 min at 37°C in imaging buffer containing the indicated ATP/ATP analog and inhibitor conditions. For time-lapse measurements, cells were permeabilized with digitonin in a Mg^2+^ free ICM buffer supplemented with 150 μM EDTA to passively deplete ER-luminal free Mg^2+^. With and without 50 μM Mg-ATP was added followed by a 20 mM MgCl_2_ pulse, to study _ER_Mg^2+^ uptake while ATP/analogs/inhibitors were maintained throughout. Confocal FRET imaging was performed at 37°C on a Leica TCS SP8 equipped with a 63× oil immersion objective and FRET-SE detection module. Excitation was at 405 nm, with emissions collected for cerulean (460 to 490 nm) and citrine (510 to 550 nm). FRET ratios were extracted using Leica FRET-SE software optimized for live-cell quantitative FRET analysis. Traces show the mean-time course for individual fields; bar graphs summarize Mg^2+^ uptake quantified under each condition.

### ERMA-SERCA1 TM substitution reveals nonredundant determinants of ER Mg^2+^ selectivity

To identify domains guiding Mg^2+^ selectivity, ERMA-SERCA1 chimeric constructs were generated by replacing individual TM helices of ERMA with the corresponding segments from the SERCA (SERCA1). Primary hepatocytes were isolated from *Erma^fl/fl^* mice using a two-step collagenase perfusion method. After 6 hours of plating, cells were transduced with Cre recombinase (Ad-iCre) to excise the endogenous ERMA gene. Cells were then transfected with WT ERMA or individual ERMA-SERCA1 chimeras, and expression was confirmed by RFP fluorescence. At 12 hours posttransfection, hepatocytes were further transduced with MagFRET-1_ER_ to enable real-time monitoring of _ER_Mg^2+^ dynamics. After 72 hours, _ER_Mg^2+^ flux was assessed by live-cell Ad-MagFRET-1_ER_ imaging in response to lactate stimulation, capturing both _ER_Mg^2+^ depletion and ERMA-mediated Mg^2+^ uptake. Confocal FRET imaging was performed at 37°C on a Leica TCS SP8 equipped with a 63× oil immersion objective and FRET-SE detection module. Excitation was at 405 nm, with emissions collected for cerulean (460 to 490 nm) and citrine (510 to 550 nm). FRET ratios were extracted using Leica FRET-SE software optimized for live-cell quantitative FRET analysis. Traces show the mean-time course for individual fields; bar graphs summarize Mg^2+^ uptake quantified under each condition.

### Quantification and statistical analysis

All data are expressed as means ± SEM unless otherwise indicated. Statistical analysis was done using a two-tailed Student’s unpaired *t* test for one or multiple samples and one-way analysis of variance (ANOVA) with Tukey’s multiple comparisons or regression/correlation analysis when required. Statistically significant was defined as *P* < 0.05. All experiments were conducted at least three times unless specified otherwise. GraphPad Prism 10 was used for statistical testing. Biorender, GraphPad Prism, and Canvas 11 were used to generate graphs and plot the figures.
