# Supplementary material for: Structural and mutational insights define ERMA as the ER Mg2+ ATPase and reservoir gatekeeper
Source: Sci Adv. 2026 Jul 1;12(27):eaef4971. doi: 10.1126/sciadv.aef4971 (PMC13322241; doi:10.1126/sciadv.aef4971)
Supplement: Supplementary file 1 — Figs. S1 to S7 Table S1 [file sciadv.aef4971_sm.pdf]

Science Advances
AAAS

# Supplementary Materials for

## Structural and mutational insights define ERMA as the ER $Mg^{2+}$ ATPase and reservoir gatekeeper

Manigandan Venkatesan *et al.*

Corresponding author: Ravi C. Kalathur, ravi.kalathur@stjude.org;
Youxing Jiang, youxing.jiang@utsouthwestern.edu; Muniswamy Madesh, muniswamy@uthscsa.edu

*Sci. Adv.* **12**, eaef4971 (2026)
DOI: 10.1126/sciadv.aef4971

**This PDF file includes:**

Figs. S1 to S7
Table S1

**Fig. S1**

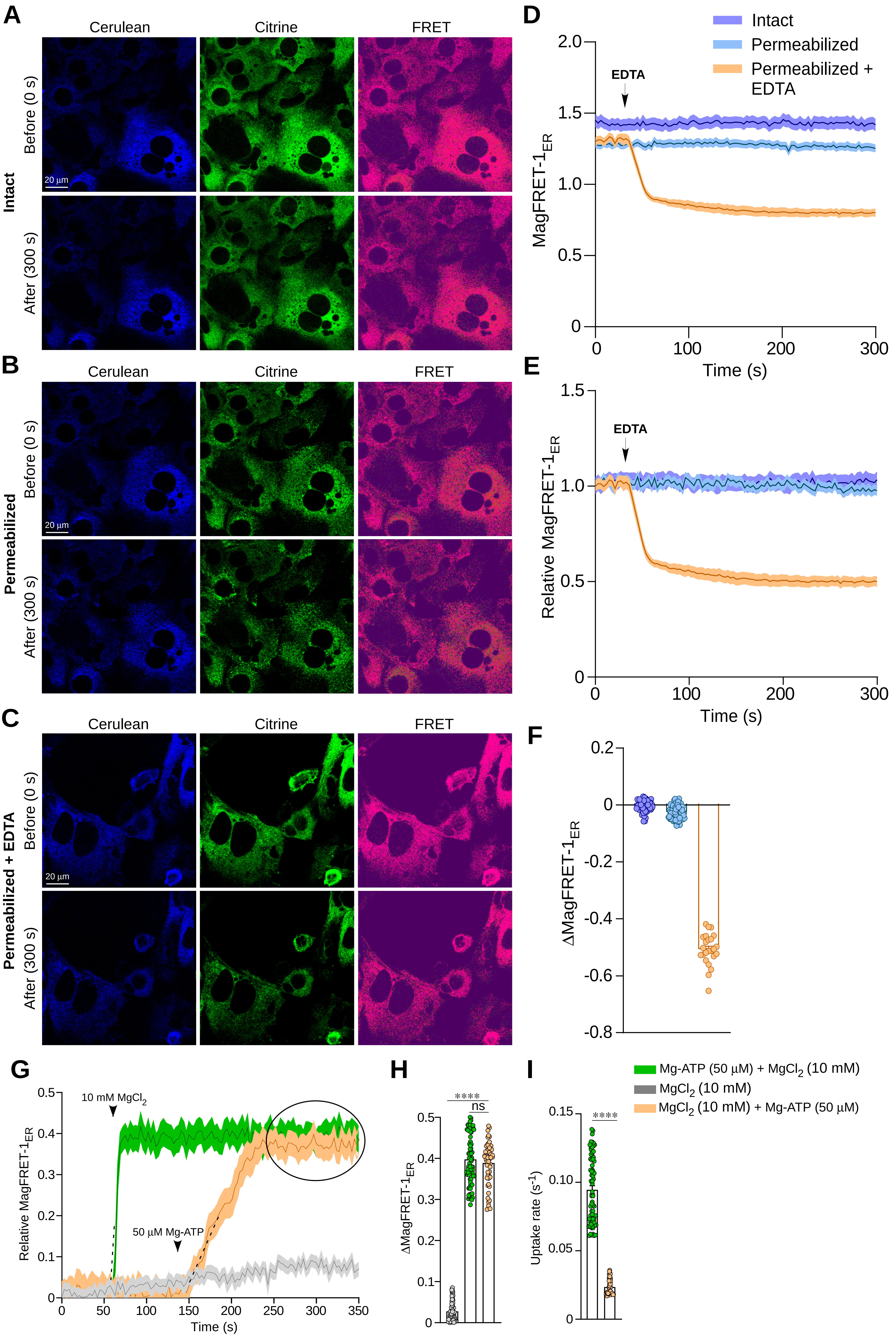

A
Cerulean
Citrine
FRET
Intact
Before (0 s)
After (300 s)
20 µm
B
Permeabilized
C
Permeabilized + EDTA
D
Intact
Permeabilized
Permeabilized + EDTA
EDTA
MagFRET-1ER
Time (s)
E
Relative MagFRET-1ER
F
ΔMagFRET-1ER
G
10 mM MgCl2
50 µM Mg-ATP
Relative MagFRET-1ER
Time (s)
H
****
ns
ΔMagFRET-1ER
I
****
Uptake rate (s-1)
Mg-ATP (50 µM) + MgCl2 (10 mM)
MgCl2 (10 mM)
MgCl2 (10 mM) + Mg-ATP (50 µM)

**Fig. S1. Development of assays to measure luminal ER Mg2+ concentration and active ER Mg2+ uptake.** (**A-C**) Representative confocal images of primary hepatocytes expressing MagFRET-1ER under intact (A), permeabilized (B), and (C) permeabilized + EDTA conditions at 0 s and 300 s. Cerulean (donor), Citrine (acceptor), and FRET channels are shown. Scale bars, 20 mm. (**D-E**) Time-resolved traces of (D) unnormalized MagFRET-1ER and (E) normalized MagFRET-1ER in intact, permeabilized, and permeabilized + EDTA conditions. (**F**) Quantification of DMagFRET-1ER responses. Data are mean ± SEM. n = 3 independent experiments and 3-5 replicates. (**G**) Representative time-resolved traces of relative MagFRET-1ER signal following the addition of 10 mM MgCl2 in the presence of pre-supplemented Mg-ATP (green trace), addition of 50 μM Mg-ATP in the presence of pre-supplemented 10 mM MgCl2 (orange trace), or addition of 10 mM MgCl2 alone (gray trace). Data are mean ± SEM. n = 3 independent experiments. (**H**) Quantification of DMagFRET-1ER signals at the indicated time points (circle as shown in panel G). Data are mean ± SEM. n = 3 independent experiments and 3-5 replicates. (**I**) Calculated rates of ERMg2+ uptake ($s^{-1}$) derived from initial slopes of MagFRET-1ER traces (dotted lines as shown in panel G). ****P < 0.0001; n.s., not significant. Data are mean ± SEM. n = 3 independent experiments and 3-5 replicates.

# Fig. S2

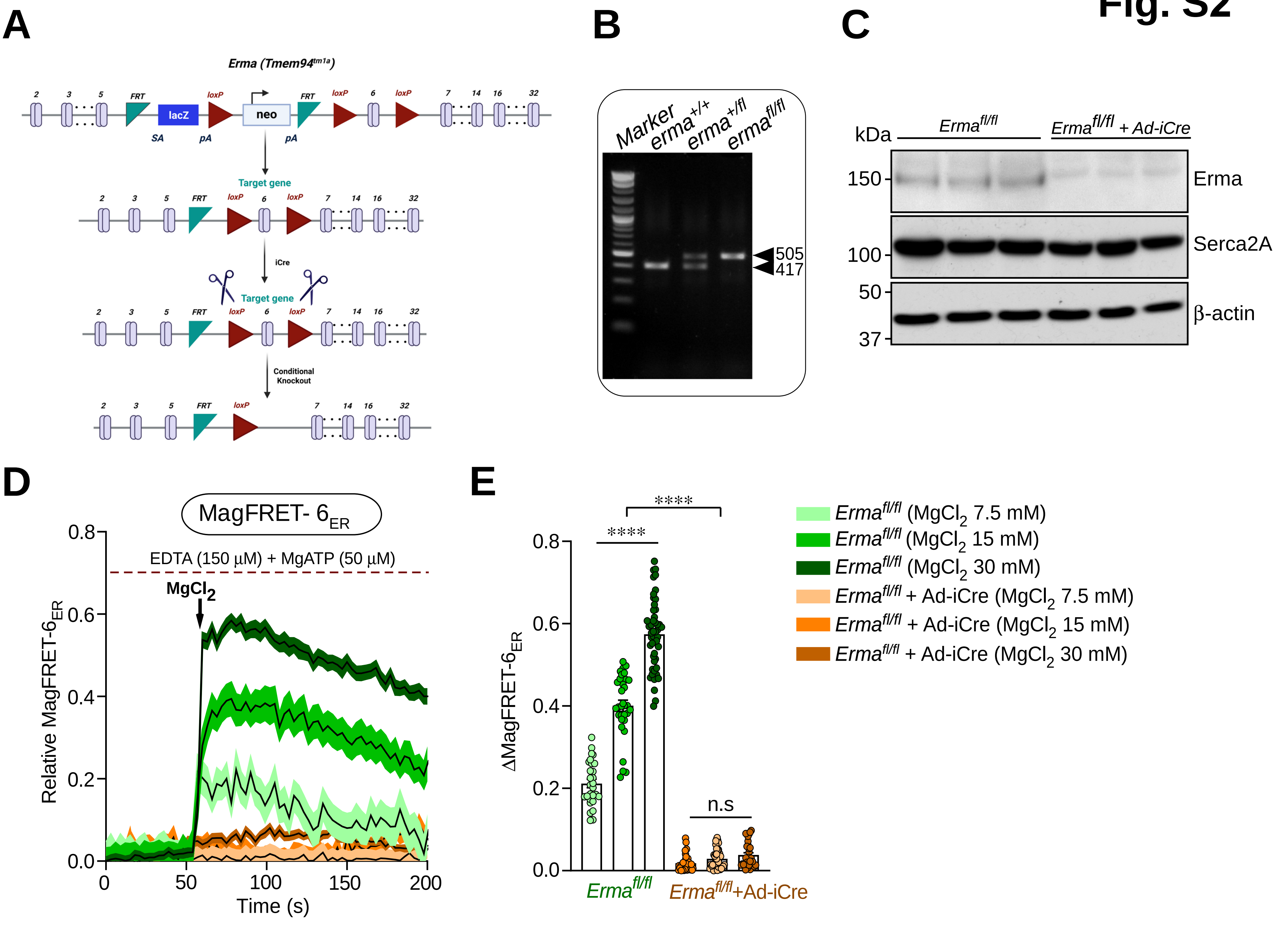
A
Erma (Tmem94tm1a)
FRT
lacZ
loxP
neo
SA
pA
Target gene
iCre
Conditional Knockout
B
Marker
erma+/+
erma+/fl
ermafl/fl
505
417
C
kDa
Ermafl/fl
Ermafl/fl + Ad-iCre
150
100
50
37
Erma
Serca2A
β-actin
D
MagFRET- 6ER
EDTA (150 µM) + MgATP (50 µM)
MgCl2
Relative MagFRET-6ER
Time (s)
E
ΔMagFRET-6ER
****
n.s
Ermafl/fl
Ermafl/fl+Ad-iCre
Ermafl/fl (MgCl2 7.5 mM)
Ermafl/fl (MgCl2 15 mM)
Ermafl/fl (MgCl2 30 mM)
Ermafl/fl + Ad-iCre (MgCl2 7.5 mM)
Ermafl/fl + Ad-iCre (MgCl2 15 mM)
Ermafl/fl + Ad-iCre (MgCl2 30 mM)

**Fig. S2. ERMA drives ER Mg2+ uptake revealed by MagFRET-6ER calibration in Ermafl/fl and Ermafl/fl + Ad-iCre hepatocytes.** (**A**) Schematic representation of the sequential steps involved in the generation of the conditional Erma allele. The Tmem94tm1a “knockout-first” allele was obtained from the EUCOMM (European Conditional Mouse Mutagenesis) program. The targeted allele contains a promoter-driven lacZ reporter and neo selection cassette flanked by FRT sites, and a critical exon (exon 6) flanked by loxP sites. The resulting floxed allele allows Cre recombinase-dependent excision of the floxed region, resulting in deletion of exon 6 and enabling tissue-specific or inducible deletion of Erma for functional studies. "Created in BioRender. Venkatesan, M. (2026) https://BioRender.com/m730uwu”. (**B**) Representative agarose gel electrophoresis showing PCR amplification products from mouse tail genomic DNA used for Erma genotyping. The WT (+) allele produces a 417 bp band, whereas the floxed (fl) allele produces a 505 bp band. Homozygous floxed (Ermafl/fl) mice display only the 505 bp band, heterozygous (Erma+/fl) mice show both 417 bp and 505 bp bands, and WT (Erma+/+) mice exhibit only the 417 bp product. DNA marker (lane 1) was used as a molecular weight reference. (**C**) Primary hepatocytes isolated from Ermafl/fl mice were infected with adenoviral iCre (Ad-iCre) to induce hepatocyte-specific recombination. Cell lysates were analyzed by immunoblotting using a custom-made rabbit polyclonal ERMA antibody, with SERCA2A and b-actin serving as loading controls. n = 3 independent experiments. (**D** and **E**) Representative traces showing normalized MagFRET-6ER responses in murine hepatocytes isolated from Ermafl/fl mice, either untreated or transduced with Ad-iCre to induce Cre-mediated excision, effectively knocking out Erma expression. After 72 h, ERMg2+ uptake was assessed using the MagFRET-6ER sensor by stepwise MgCl2 additions (7.5, 15, and 30 mM). Quantification of Mg2+ uptake as MagFRET-6ER under the indicated conditions. Data represent mean ± SEM from n = 3 independent experiments. (n.s.= not significant; ****p < 0.0001).

# Fig. S3

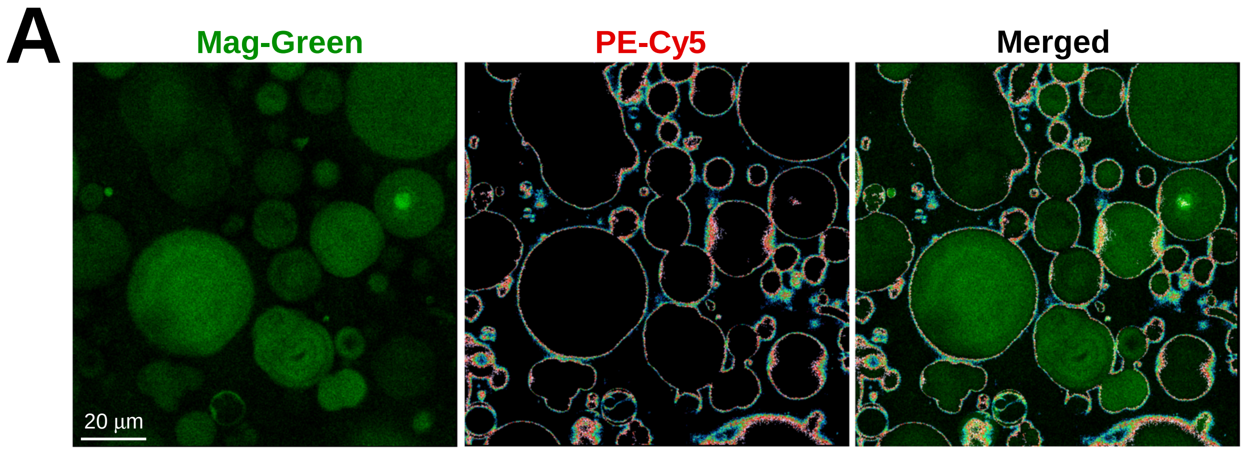
A
Mag-Green
PE-Cy5
Merged
20 µm

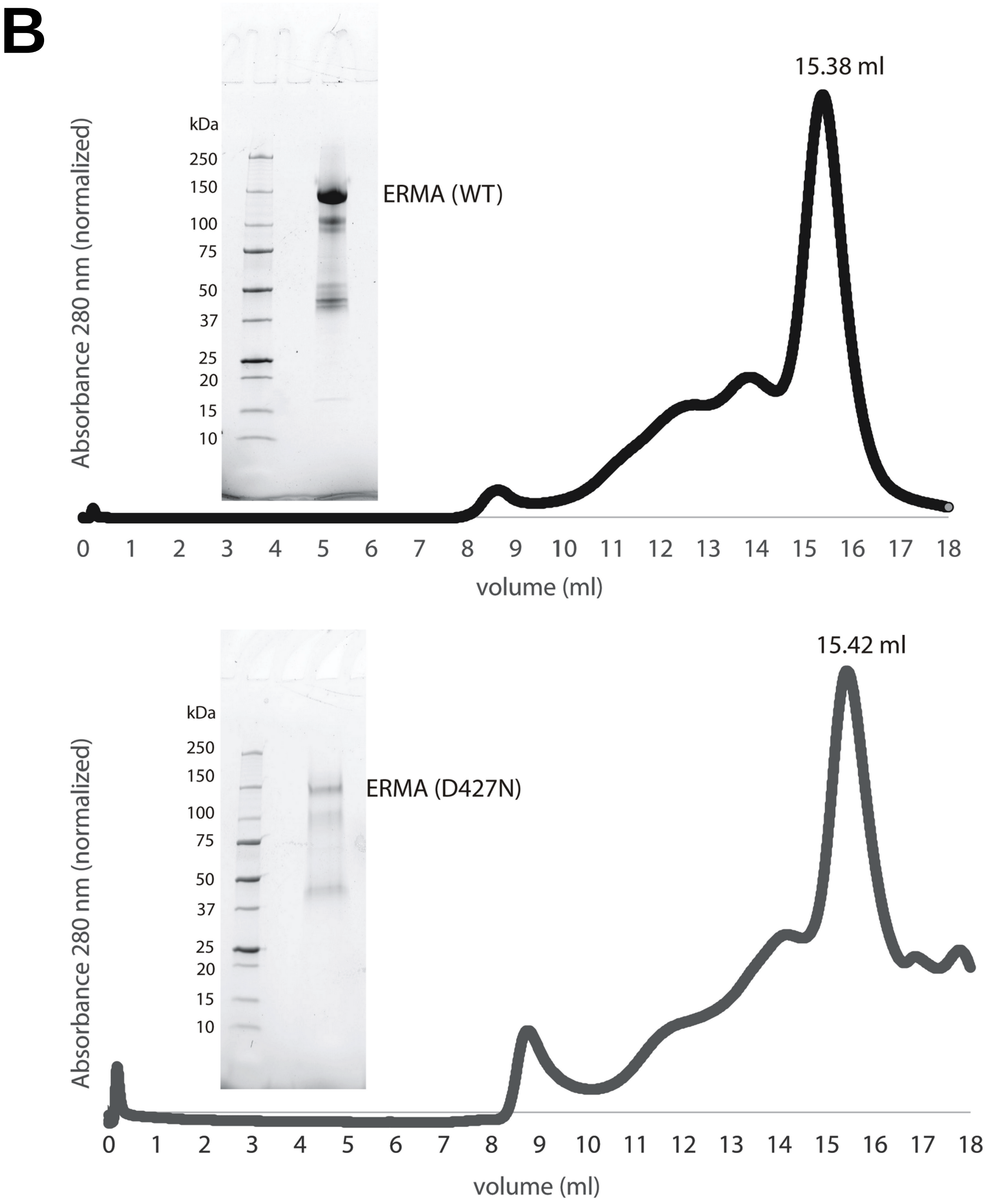
B
15.38 ml
kDa
250
150
100
75
50
37
25
20
15
10
ERMA (WT)
Absorbance 280 nm (normalized)
0 1 2 3 4 5 6 7 8 9 10 11 12 13 14 15 16 17 18
volume (ml)
15.42 ml
kDa
250
150
100
75
50
37
25
20
15
10
ERMA (D427N)
Absorbance 280 nm (normalized)
0 1 2 3 4 5 6 7 8 9 10 11 12 13 14 15 16 17 18
volume (ml)

**Fig. S3. ERMA reconstitution into proteoliposomes and biochemical characterization of WT and D427N mutant.** (**A**) Representative confocal images of proteoliposomes reconstituted with purified ERMA and labelled with Mag-Green (green) for Mg2+ sensing and PE-Cy5 (red) to visualize lipid membranes. The merged image demonstrates proper membrane incorporation and uniform distribution of ERMA within the liposomal bilayer. Scale bar: 20 μm. (**B**) Size-exclusion chromatograms of purified recombinant human ERMA (WT) and the ERMA (D427N) catalytic-site mutant. Both proteins eluted as monodisperse peaks at ~15.4 ml, consistent with a stable oligomeric assembly. Inset SDS-PAGE analyses confirm comparable expression and purity for WT and D427N preparations used for reconstitution and downstream functional assays.

Fig. S4

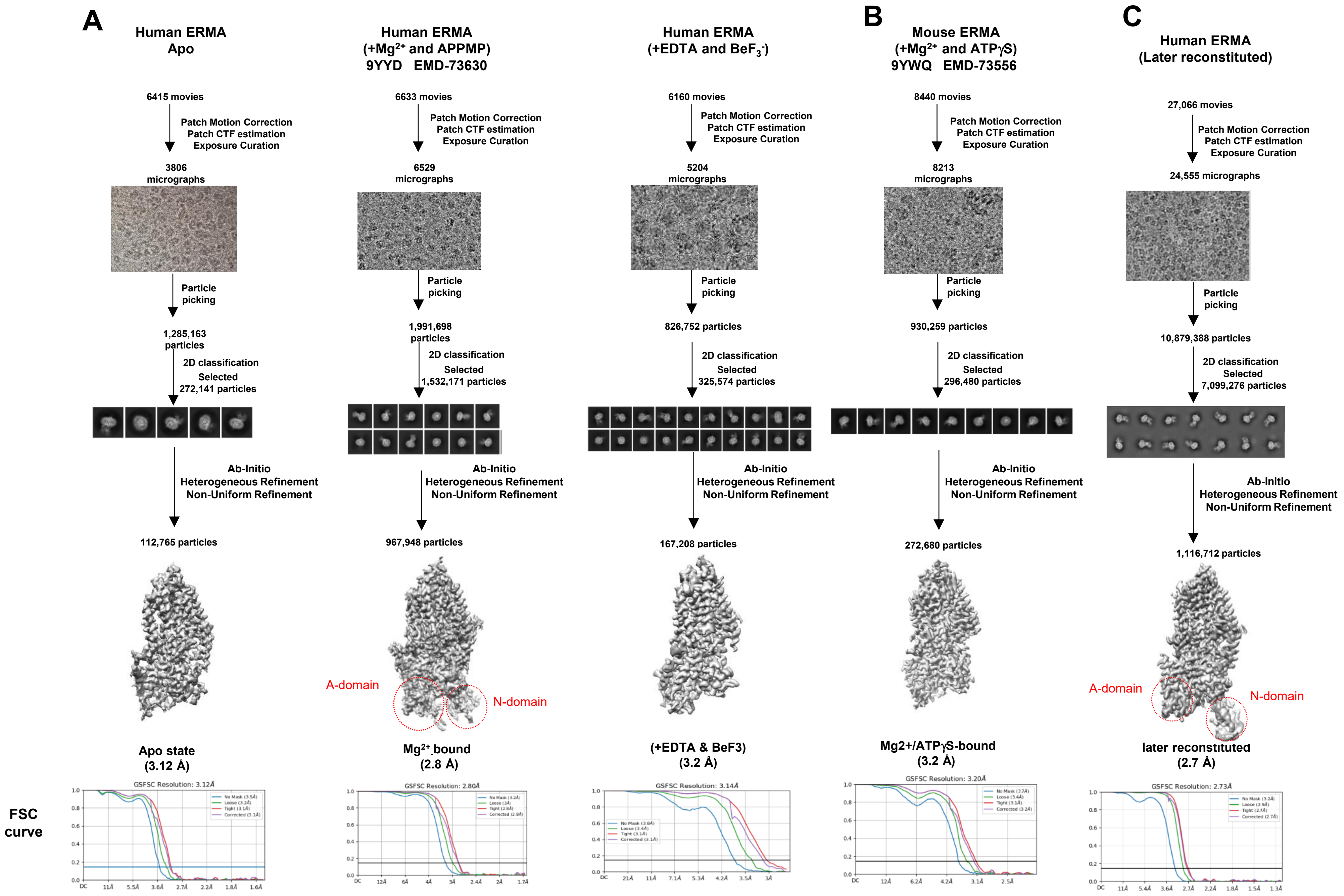
A
Human ERMA
Apo
6415 movies
Patch Motion Correction
Patch CTF estimation
Exposure Curation
3806
micrographs
Particle
picking
1,285,163
particles
2D classification
Selected
272,141 particles
Ab-Initio
Heterogeneous Refinement
Non-Uniform Refinement
112,765 particles
Apo state
(3.12 Å)
GSFSC Resolution: 3.12Å
Human ERMA
(+Mg2+ and APPMP)
9YYD EMD-73630
6633 movies
Patch Motion Correction
Patch CTF estimation
Exposure Curation
6529
micrographs
Particle
picking
1,991,698
particles
2D classification
Selected
1,532,171 particles
Ab-Initio
Heterogeneous Refinement
Non-Uniform Refinement
967,948 particles
A-domain
N-domain
Mg2+ bound
(2.8 Å)
GSFSC Resolution: 2.80Å
Human ERMA
(+EDTA and BeF3-)
6160 movies
Patch Motion Correction
Patch CTF estimation
Exposure Curation
5204
micrographs
Particle
picking
826,752 particles
2D classification
Selected
325,574 particles
Ab-Initio
Heterogeneous Refinement
Non-Uniform Refinement
167,208 particles
(+EDTA & BeF3)
(3.2 Å)
GSFSC Resolution: 3.14Å
B
Mouse ERMA
(+Mg2+ and ATPγS)
9YWQ EMD-73556
8440 movies
Patch Motion Correction
Patch CTF estimation
Exposure Curation
8213
micrographs
Particle
picking
930,259 particles
2D classification
Selected
296,480 particles
Ab-Initio
Heterogeneous Refinement
Non-Uniform Refinement
272,680 particles
Mg2+/ATPγS-bound
(3.2 Å)
GSFSC Resolution: 3.20Å
C
Human ERMA
(Later reconstituted)
27,066 movies
Patch Motion Correction
Patch CTF estimation
Exposure Curation
24,555 micrographs
Particle
picking
10,879,388 particles
2D classification
Selected
7,099,276 particles
Ab-Initio
Heterogeneous Refinement
Non-Uniform Refinement
1,116,712 particles
A-domain
N-domain
later reconstituted
(2.7 Å)
GSFSC Resolution: 2.73Å
FSC
curve

# Fig. S4

D

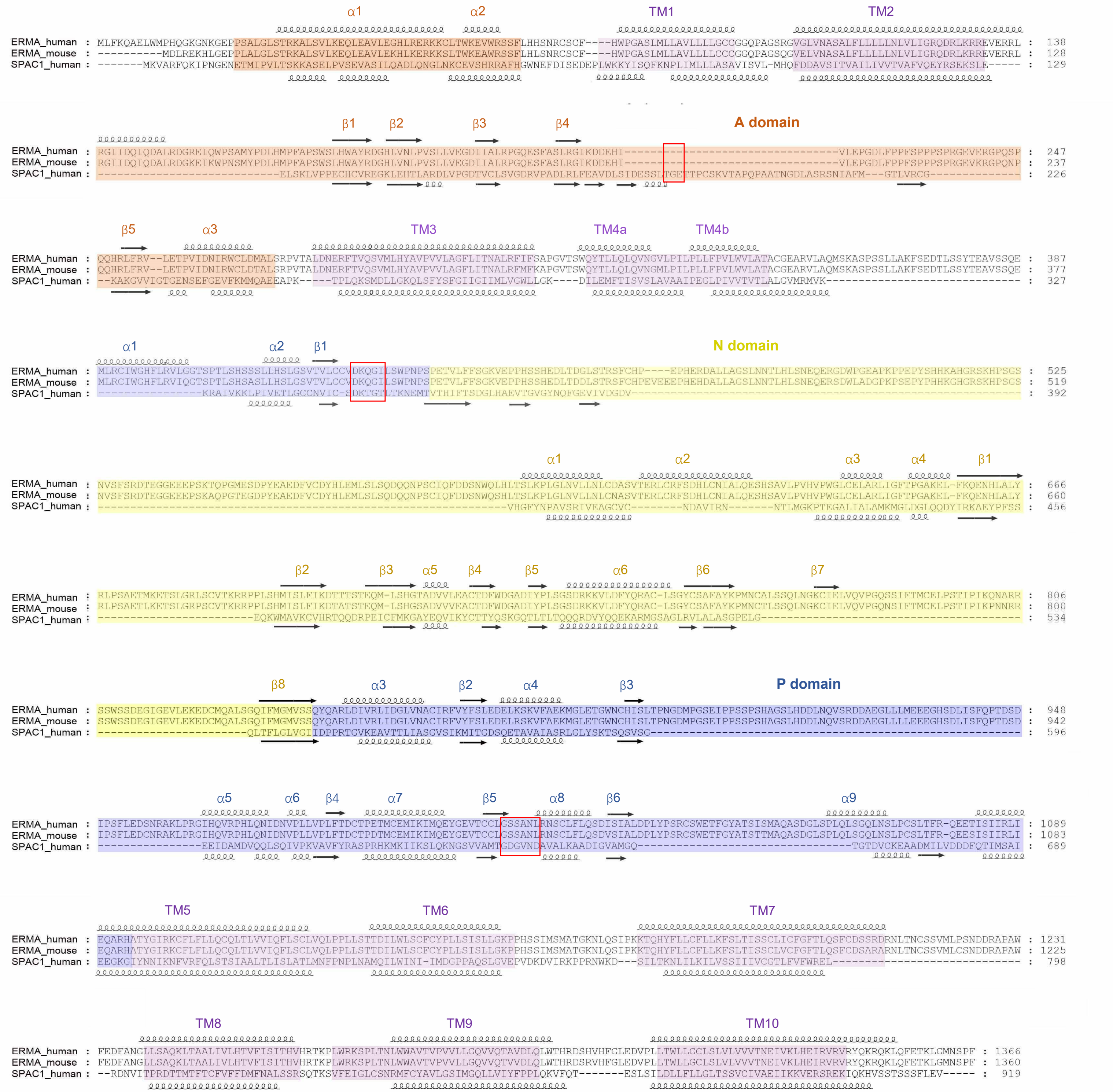
A domain
N domain
P domain
TM1
TM2
TM3
TM4a
TM4b
TM5
TM6
TM7
TM8
TM9
TM10
ERMA_human
ERMA_mouse
SPAC1_human

**Fig. S4. Cryo-EM data processing scheme for the human and mouse ERMA structures.** Representative image processing pipelines used to obtain high-resolution cryo-EM reconstructions of ERMA under distinct biochemical states. For each dataset, the numbers of collected movies, curated micrographs, picked particles, and selected 2D classes are shown, followed by the refinement strategy (ab-initio modeling, heterogeneous refinement, and non-uniform refinement). Final particle stacks and resulting 3D reconstructions are displayed for: (**A**) Human ERMA in the apo conformation (3.12 Å), human ERMA in the presence of Mg2+ and the non-hydrolyzable ATP analog AMPPCP (2.8 Å). Human ERMA in the presence of EDTA and BeF3 (3.14 Å). (**B**) Mouse Erma in Mg2+/ATPg-bound form (3.2 Å). (**C**) Later reconstituted human ERMA with Mg2+/AMPPCP (2.7 Å). Fourier shell correlation (FSC) plots demonstrate final map resolutions at the gold-standard FSC = 0.143 criterion. Conformational variability of the A-domain and N-domain is indicated for nucleotide-bound reconstructions. (**D**) Structure-based alignment between ERMA and SPAC1. The secondary structure assignments above the sequence are based on the structure of human ERMA. I ndividual domains are highlighted in different colors, and their secondary structures are assigned individually. The secondary structure assignments below the sequence are based on the structure of human SPAC1 (PDB: 8IWR). Key sequence motifs important for phosphorylation and dephosphorylation in P-type ATPases are boxed in red.

Fig. S5

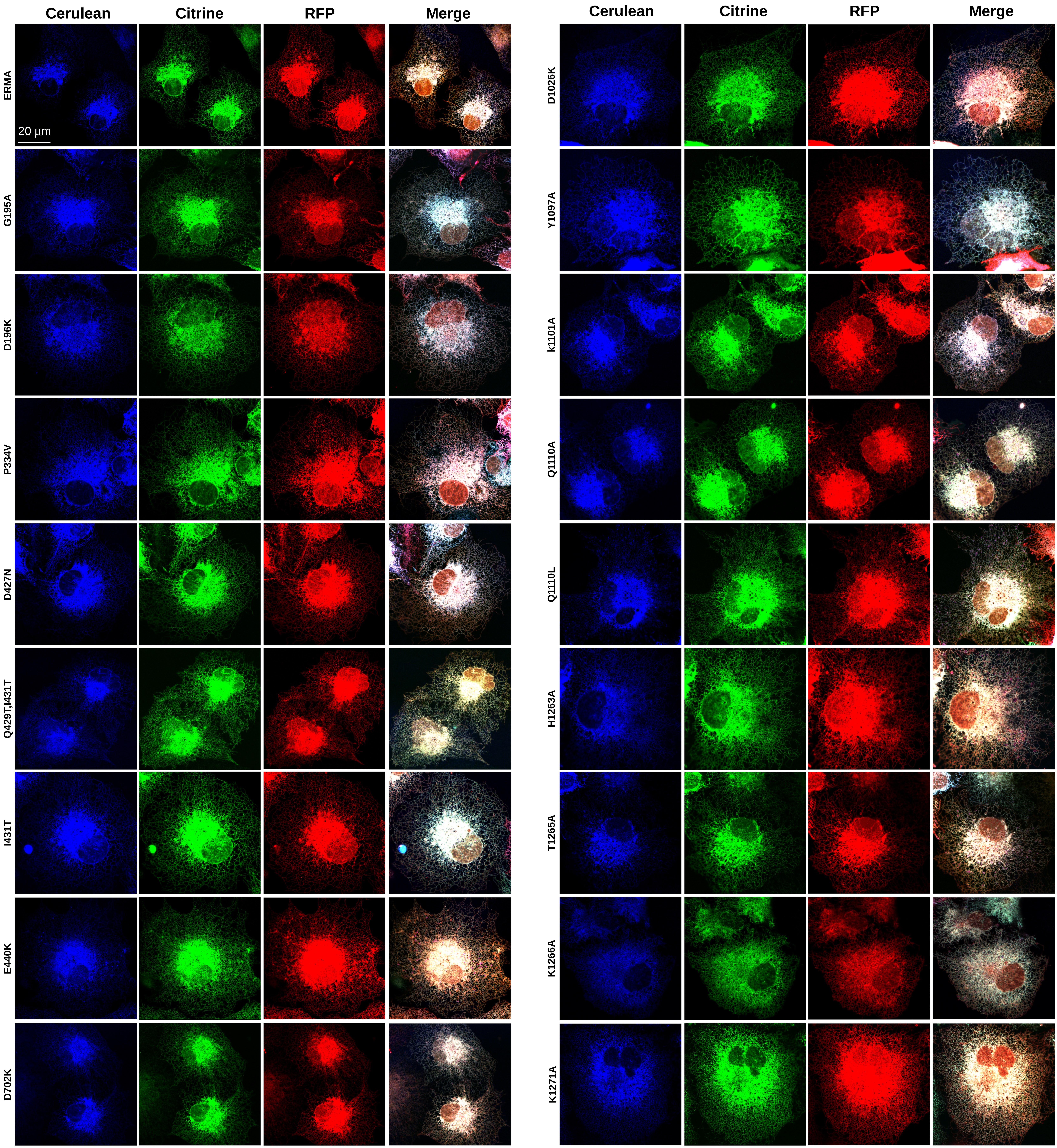
Cerulean
Citrine
RFP
Merge
ERMA
20 μm
G195A
D196K
P334V
D427N
Q429T,I431T
I431T
E440K
D702K
Cerulean
Citrine
RFP
Merge
D1026K
Y1097A
K1101A
Q1110A
Q1110L
H1263A
T1265A
K1266A
K1271A

**Fig. S5. ERMA mutant constructs demonstrate proper localization within the ER compartment.** Representative confocal microscopic images of COS-7 cells co-expressing the adenoviral MagFRET-1ER sensor with either human ERMA-mRFP or ERMA mutant mRFP constructs. The subcellular distribution of ERMA and its mutants was visualized by confocal microscopy to assess targeting and expression. Scale bar, 20 μm. Data shown are representative of n = 3 independent experiments.

# Fig. S6

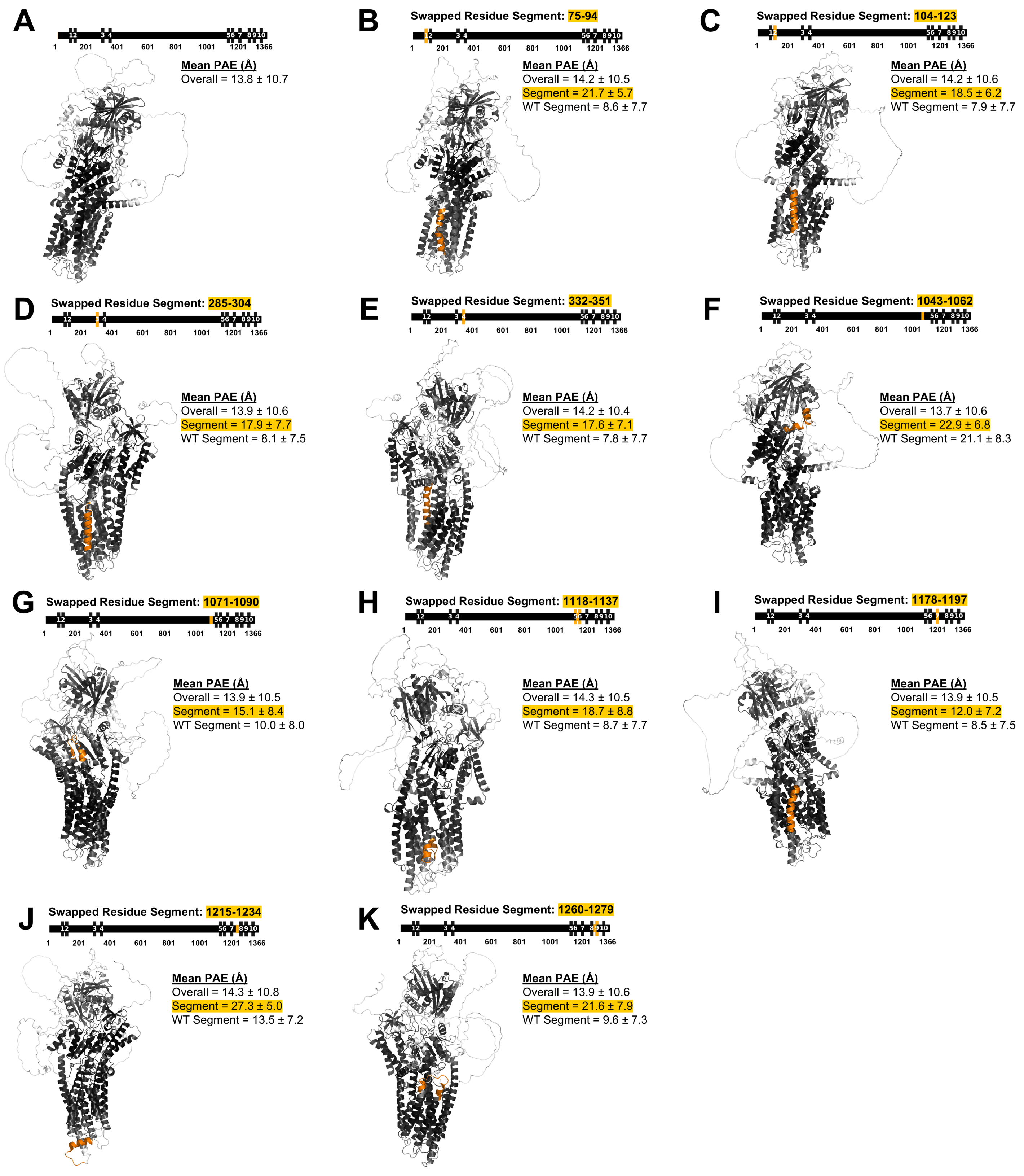
A
Mean PAE (Å)
Overall = 13.8 ± 10.7
B
Swapped Residue Segment: 75-94
Mean PAE (Å)
Overall = 14.2 ± 10.5
Segment = 21.7 ± 5.7
WT Segment = 8.6 ± 7.7
C
Swapped Residue Segment: 104-123
Mean PAE (Å)
Overall = 14.2 ± 10.6
Segment = 18.5 ± 6.2
WT Segment = 7.9 ± 7.7
D
Swapped Residue Segment: 285-304
Mean PAE (Å)
Overall = 13.9 ± 10.6
Segment = 17.9 ± 7.7
WT Segment = 8.1 ± 7.5
E
Swapped Residue Segment: 332-351
Mean PAE (Å)
Overall = 14.2 ± 10.4
Segment = 17.6 ± 7.1
WT Segment = 7.8 ± 7.7
F
Swapped Residue Segment: 1043-1062
Mean PAE (Å)
Overall = 13.7 ± 10.6
Segment = 22.9 ± 6.8
WT Segment = 21.1 ± 8.3
G
Swapped Residue Segment: 1071-1090
Mean PAE (Å)
Overall = 13.9 ± 10.5
Segment = 15.1 ± 8.4
WT Segment = 10.0 ± 8.0
H
Swapped Residue Segment: 1118-1137
Mean PAE (Å)
Overall = 14.3 ± 10.5
Segment = 18.7 ± 8.8
WT Segment = 8.7 ± 7.7
I
Swapped Residue Segment: 1178-1197
Mean PAE (Å)
Overall = 13.9 ± 10.5
Segment = 12.0 ± 7.2
WT Segment = 8.5 ± 7.5
J
Swapped Residue Segment: 1215-1234
Mean PAE (Å)
Overall = 14.3 ± 10.8
Segment = 27.3 ± 5.0
WT Segment = 13.5 ± 7.2
K
Swapped Residue Segment: 1260-1279
Mean PAE (Å)
Overall = 13.9 ± 10.6
Segment = 21.6 ± 7.9
WT Segment = 9.6 ± 7.3
1 201 401 601 801 1001 1201 1366

Fig. S6

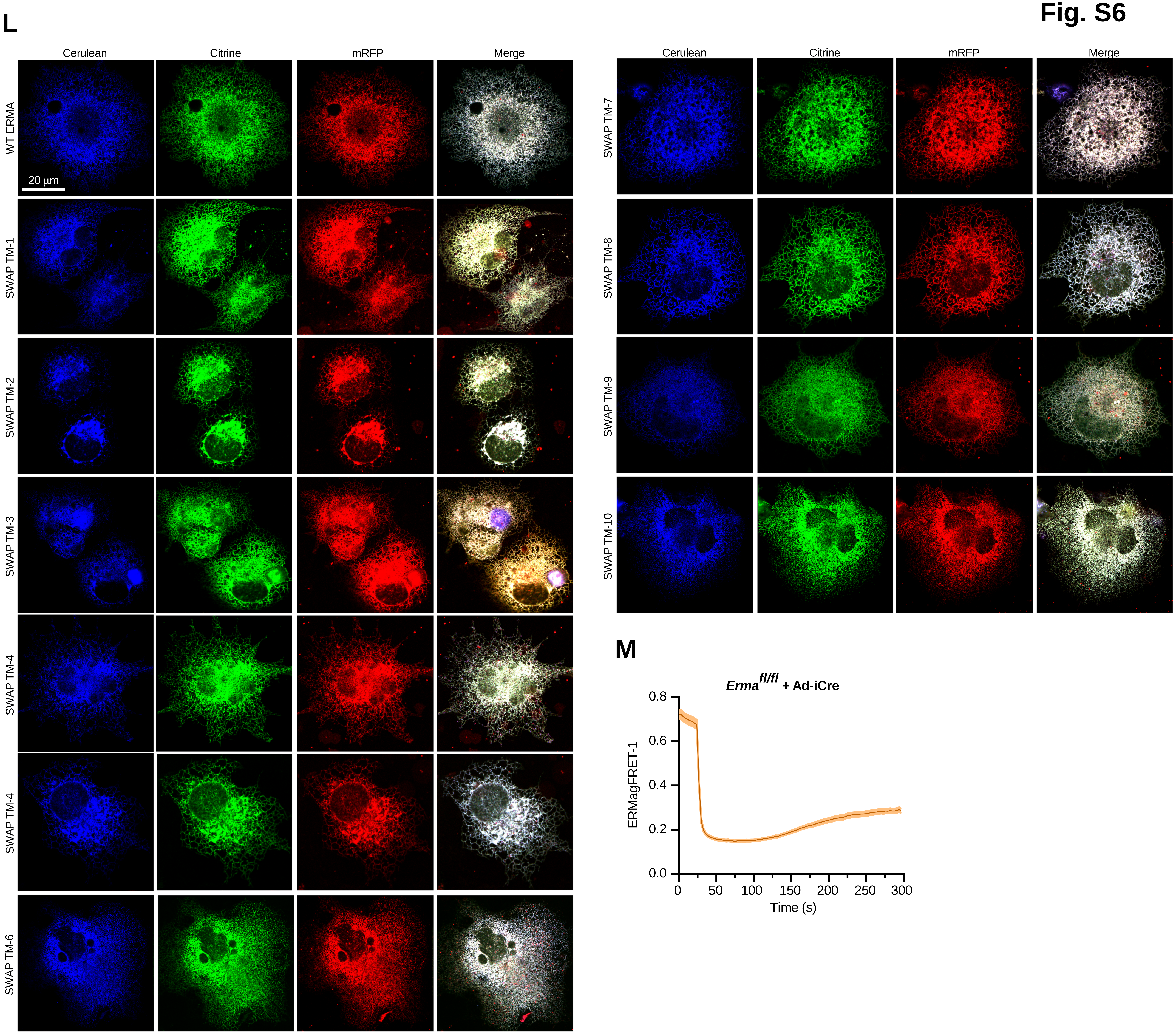
L
Cerulean
Citrine
mRFP
Merge
WT ERMA
20 µm
SWAP TM-1
SWAP TM-2
SWAP TM-3
SWAP TM-4
SWAP TM-4
SWAP TM-6
SWAP TM-7
SWAP TM-8
SWAP TM-9
SWAP TM-10
M
Erma fl/fl + Ad-iCre
ERMagFRET-1
0.8
0.6
0.4
0.2
0.0
0
50
100
150
200
250
300
Time (s)

**Fig. S6. AlphaFold3 predictions and expression analysis of ERMA-SERCA1 chimeric constructs demonstrate proper localization of mutant chimeras within the ER compartment.** (**A**) AlphaFold3 prediction of human wild-type (WT) ERMA. At top, a domain architecture diagram highlights the UniProt-predicted locations of the transmembrane (TM) regions, numbered 1-10, with corresponding residue numbers shown below the architecture. (**B-K**) Structural predictions of ERMA-SERCA1a chimeras, swapping (B) ERMA residues 75-94 with SERCA1 50-69, (C) ERMA residues 104-123 with SERCA1 91-110, (D) ERMA residues 285-304 with SERCA1 194-213, (E) ERMA residues 332-351 with SERCA1 296-315, (F) ERMA residues 1043-1062 with SERCA 758-777, (G) ERMA residues 1071-1090 with SERCA1 788-808, (H) ERMA residues 1118-1137 with SERCA1 829-851, (I) ERMA residues 1178-1197 with SERCA1 898-917, (J) ERMA residues 1215-1234 with SERCA1 residues 931-949 and (K) ERMA residues 1260-1279 with SERCA residues 965-985. In B-K, FASTA UniProt sequences Q12767-3 (ERMA) and O14983 (SERCA1a) were used for the model generation, and the domain-swapped region is coloured orange in the domain architecture and the AlphaFold predicted structure. In A-K, the overall mean PAE ± SD, mean PAE ± SD of the swapped residue segments relative to all other residues and the mean PAE ± SD of the homologous WT segment relative to all other residues are indicated. All backbone three-dimensional (3D) structures are colored as a gradient from white to black, corresponding to predicted local distance differences test (pLDDT) scores from 0 to 70, respectively, and are oriented with the cytosolic domains at top and TM domains at bottom. (**L**) Representative confocal microscopic images of COS-7 cells co-expressing the adenoviral MagFRET-1ER sensor with either human ERMA-mRFP or individual ERMA-SERCA1 transmembrane (TM) chimera swap (TM1 - TM10) -mRFP constructs. The subcellular distribution of ERMA and its chimeras was visualized by confocal microscopy to assess targeting and expression. Scale bar, 20 µm. Data shown are representative of n = 3 independent experiments. (**M**) Representative MagFRET-1ER mean traces of Ermafl/fl + Ad-iCre treated hepatocytes signal after 5 mM Lactate stimulation.

# Fig. S7

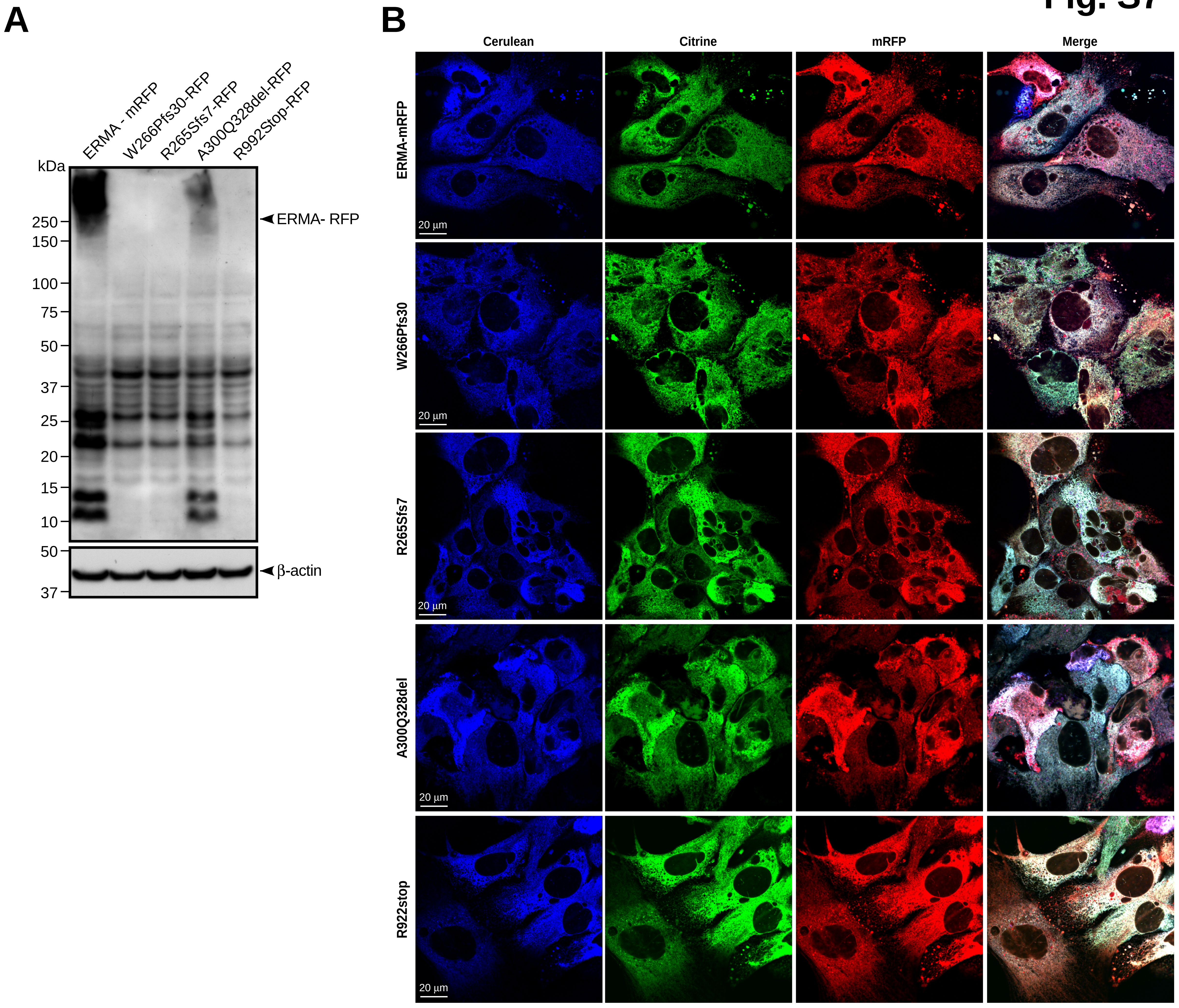
A
B
ERMA - mRFP
W266Pfs30-RFP
R265Sfs7-RFP
A300Q328del-RFP
R992Stop-RFP
kDa
250
150
100
75
50
37
25
20
15
10
50
37
ERMA- RFP
β-actin
Cerulean
Citrine
mRFP
Merge
ERMA-mRFP
W266Pfs30
R265Sfs7
A300Q328del
R922stop
20 μm

**Fig. S7. Expression and subcellular localization of human pathogenic ERMA mutant constructs in WT hepatocytes.** (**A**) Immunoblot analysis of ERMA-mRFP and human pathogenic variants (W266Pfs30, R265Sfs7, A300Q328del, and R922stop). β-actin serves as a loading control. (**B**) Representative confocal images of cells co-expressing MagFRET-1ER sensor (Cerulean/Citrine) and mRFP-tagged ERMA constructs (WT and mutants). Merge shows the overlap of signals. Data shown are representative of n = 3 independent experiments.

# Supplementary Table 1: Cryo-EM data collection, refinement and validation statistics

| | hERMA Apo | hERMA $Mg^{2+}$ + AMPPCP EMD-73630 PDB-9YYD | hERMA EDTA + $BeF_3^-$ | mERMA $Mg^{2+}$ +ATPγS EMD-73556 PDB-9YWQ | hERMA $Mg^{2+}$ + AMPPCP (Later reconstituted) |
|---|---|---|---|---|---|
| **Data collection and processing** | | | | | |
| Magnification | 165,000 | 105,000 | 130,000 | 165,000 | 130,000 |
| Voltage (kV) | 300 | 300 | 300 | 300 | 300 |
| Electron exposure ($e^-$/Å$^2$) | 60 | 60 | 60 | 60 | 64 |
| Defocus range (μm) | -0.9 - -2.2 | -0.9 - -2.2 | -0.9 - -2.2 | -0.9 - -2.2 | -0.5 - -2.5 |
| Pixel size (Å) | 0.738 | 0.827 | 0.664 | 0.51 | 0.649 |
| Symmetry imposed | C1 | C1 | C1 | C1 | C1 |
| Initial particle images (no.) | 1,596,675 | 1,991,698 | 826,752 | 930,259 | 10,879,388 |
| Final particle images (no.) | 532,383 | 967,948 | 167,208 | 272,680 | 1,116,712 |
| Map resolution (Å) | 3.12 | 2.8 | 3.2 | 3.2 | 2.7 |
| FSC threshold: 0.143 | | | | | |
| **Refinement** | | | | | |
| Model resolution (Å) FSC threshold: 0.143 | 2.89 | 2.75 | 3.2 | 3.19 | 2.73 |
| Map sharpening B factor (Å$^2$) | -150.7 | -133.9 | -123.5 | -138.8 | -127.9 |
| Model composition | | | | | |
| Non-hydrogen atoms | 6453 | 8520 | 6507 | 6676 | 7458 |
| Protein residues | 810 | 1077 | 818 | 825 | 939 |
| Ligands | PEV 1 | $Mg^{2+}$: 1 PEV 1 | PEV 1 | AGS 1 Mg2+ 1 | PEV 1 |
| B factors (Å$^2$) | | | | | |
| Protein | 163.75 | 218.19 | 248.31 | 174.97 | 134.42 |
| Ligand | 111.64 | 86.78 | 144.29 | 116.41 | 136.98 |
| R.m.s. deviations | | | | | |
| Bond lengths (Å) | 0.002 | 0.002 | 0.003 | 0.002 | 0.002 |
| Bond angles (°) | 0.458 | 0.454 | 0.489 | 0.496 | 0.528 |
| Validation | | | | | |
| MolProbity score | 1.3 | 1.73 | 1.57 | 1.51 | 1.48 |
| Clashscore | 5.03 | 5.88 | 4.09 | 3.37 | 6.17 |
| Poor rotamers (%) | 0.28 | 2.08 | 2.33 | 1.22 | 0.00 |
| Ramachandran plot | | | | | |
| Favored (%) | 97.62 | 97.08 | 97.52 | 95.54 | 97.27 |
| Allowed (%) | 2.38 | 2.92 | 2.36 | 4.54 | 2.62 |
| Disallowed (%) | 0.00 | 0.00 | 0.12 | 0.00 | 0.11 |
